# Safety assessment of electromagnetic fields of different transmitters and receivers for EVs static charging

**DOI:** 10.1038/s41598-025-97881-9

**Published:** 2025-04-30

**Authors:** Mahmoud M. Elymany, Ahmed A. S. Mohamed, Ahmed A. Shaier, Mohamed A. Enany, Hamid Metwally, Sameh I. Selem

**Affiliations:** 1 Electrical Power and Machines Department, Faculty of Engineering, Zagazig University, Zagazig, 44519 Egypt; 2 Eaton Research Labs, Eaton Corporate, Golden, CO USA

**Keywords:** Electromagnetic fields, Electric fields, Safety, Circular (CP), Double-D (DDP), IPT, Electrical and electronic engineering, Batteries

## Abstract

This study aims to assess the safety aspect of future inductive charging stations by investigating the electromagnetic fields performance of various pad architectures. Following the recommendations of the standard {Society of Automotive Engineering (SAE J2954)}, which suggests two common pad kinds for the inductive power transfer (IPT) system (circular pad (CP) and double-D pad (DDP). The safety analysis is performed on the car side using these two types of pad architectures, with ground clearance compliant with Z3-class requirements and a power transfer of 11.1 kVA. In one scenario, a DD pad serves as the universal ground side pad (transmitter), while in the other scenario, a Circular pad is utilized. Safety assessments are performed using four models constructed based on 3D finite-element models (FEMs) and resonant networks. Circuit models are employed to establish the frequency of operation and resonant network components necessary to attain the rated transmitted power with maximum efficiency (*η*). Electric fields (*E*) and electromagnetic fields (EMFs) were calculated under ideal alignment conditions as well as in various cases of misalignment, including angular and lateral misalignments. The results demonstrate that the two distinct car side pads (CP and DDP) can function with the universal transmitter regardless of whether a CP or DDP is utilized, and that both types of car side pads (receivers) can achieve a high level of safety. Meanwhile, electric and electromagnetic fields stay within the bounds allowed by the 1998 and 2010 versions of the ICNIRP guidelines.

## Introduction

In recent years, the increasing reliance on public transportation has resulted in higher emissions of harmful gases. As a result, there is an urgent need to explore alternative transportation methods to reduce the reliance on fossil fuels in cars. One such alternative is electric vehicles (EVs), which aim to reduce fossil fuel dependence, protect the health of living beings, and minimize gas emissions and environmental damage. With the use of wireless power transfer (WPT) technology, EV batteries can be charged without the need for physical connections. This process is simple, eliminates the effort required from the driver, and operates autonomously, making it ideal for challenging environmental conditions such as rain, high temperatures, snow, and more^1^. EV batteries can be recharged through three different scenarios. The first approach allows the vehicle to replenish its battery while remaining stationary for extended durations, such as when parked in garages or parking lots. This method is referred to as the stationary or static charging scenario^2–4^. The second charging scenario involves replenishing an EV’s battery as it moves along a designated charging lane at a certain speed on highways. This method is known as in-motion charging or charging while driving^5–8^. Additionally, EVs can be charged during brief stops at traffic signals or while traveling at low speeds, a process referred to as opportunistic or semi-dynamic charging^9,10^. The concept of wireless power transmission dates back to the late nineteenth century when Hertz conducted experiments with electromagnetic waves in a vacuum, leading to its discovery^11^. Between 1894 and 1918, Nikola Tesla constructed the Tesla Tower, placing it on a high tower and encircling it with a large coil and an enormous copper ball, aiming to transmit power wirelessly through electrostatic induction^12^. From 2007 to 2013, a team of researchers from the Massachusetts Institute of Technology (MIT) carried out a series of experiments on wireless power transmission. They successfully transmitted 60 W of power over a 2-m airgap with 40% efficiency by utilizing magnetic coupling between resonators^13^. Since then, there has been an increasing interest in wireless power transmission (WPT) technology, with applications expanding to electric vehicles, smartphones, and beyond.

**Fig. 1:**
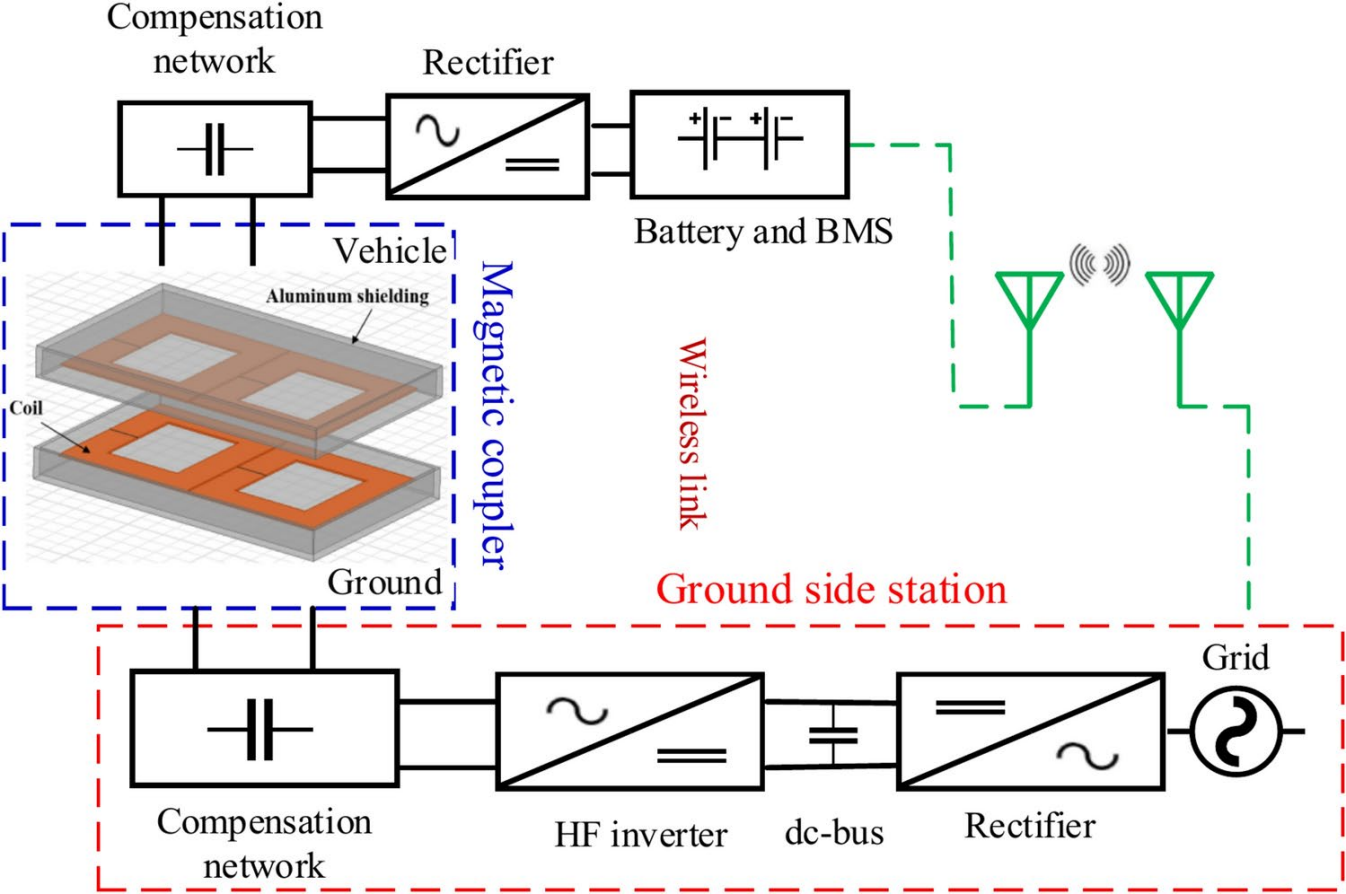
Elements for an optimal IPT system.

WPT can be categorized into four primary methods: far-field transfer (FFT)^2^, magnetic gear transfer (MGT)^14^, acoustic transfer (AT)^15^, and near-field transfer (NFT)^16^. The MGT technique utilizes permanent magnets for energy transfer, originally serving as a replacement for conventional mechanical gears. Over time, this technology has evolved and found applications in various fields, including medical devices and static EV charging^17,18^. WPT also relies on electromagnetic fields, which are classified into near-field and far-field regions. In the NFT technique, power is transmitted wirelessly through electromagnetic induction, leading to the emergence of two types of fields. The first type, known as IPT^2^, is generated by magnetic coils and is confined to a limited area around the transmission coil. The second type, capacitive power transfer (CAPT), occurs due to electric fields formed by capacitors^19,20^. IPT is an innovative technology that utilizes the magnetic fields of inductive coils to wirelessly transfer power across an air gap. With IPT, charging an EV becomes possible without the need for cables. The IPT system is an excellent option for EV charging due to its numerous advantages, such as its ability to transmit power across an air gap that meets the full ground clearance requirements (ranging from 100 to 400 mm) for all types of EVs^21^, the charging process is entirely automated, requiring no user intervention. It operates without emitting any pollutants and is designed to endure harsh weather conditions. Additionally, its absence of moving parts eliminates the need for regular maintenance. Compared to plug-in charging, this method is also safer, reducing potential hazards^22^. The IPT system consists of two main components, each comprising multiple parts. The first is the transmitter pad, which is embedded beneath the ground, and the second is the receiver pad, which is mounted on the vehicle, as illustrated in Fig. 1^2,23^.

The ground station supplies high-frequency (HF) power to the transmitter and consists of a high-frequency inverter, a DC bus, and a rectifier. The power source is initially converted from low-frequency AC electricity into DC power through rectification. The HF inverter then transforms the DC power back into high-frequency AC electricity. To ensure efficient power transmission while maintaining nominal power levels, compensating elements are used^5,24^. These compensation networks are composed of capacitors (C) or a combination of capacitors and coils (LC circuits). The transmitter coil generates electromagnetic fields that pass through the air gap and extend into the surrounding area, inducing a response in the receiving coil. When the receiver pad interacts with the varying high-frequency magnetic field, it generates an electromotive force (emf) and current (i), producing HF AC power on the vehicle side. This HF AC power is then processed through a resonant circuit and rectifier to convert it into DC power, making it suitable for charging the vehicle’s battery. To enhance power transfer and minimize the size of IPT system components, an operating frequency range between 79 and 90 kHz is preferred^25^. A communication link facilitates interaction between the ground and vehicle sides, ensuring optimal alignment, supporting billing transactions, and notifying the driver of the battery’s charge status, whether it is depleted or fully charged^26,27^. The inductive charger, which consists of both the ground and vehicle-side pads, plays a key role in the charging process by transferring power from the transmitter pad to the receiver pad and ultimately to the vehicle’s battery. Different designs and configurations of magnetic charging pads have been explored in literature. Non-polarized pads, such as rectangular pads (RP) and circular pads (CP), generate columnar magnetic flux. Meanwhile, polarized pads like bipolar pads (BP) and double-D pads (DDP) produce horizontal flux^2^. Among these configurations, the SAE J2954 standard considers CP and DDP architectures for wireless power transfer applications^28^.

WPT for EVs charging has been evolving to achieve power levels above 11 kW, enhancing charging speed and convenience through IPT and CAPT. Traditional wireless chargers, conforming to SAE J2954, support up to 11 kW, but advancements such as the Online Electric Vehicle (OLEV) system at KAIST have demonstrated 20 kW WPT over a 20 cm air gap, validating the feasibility of high-power dynamic charging^29^. Further, a 100-kW wireless charging system has been investigated for EV applications, indicating the potential of IPT technology to replace wired fast-charging stations^30^. Higher power transfer requires careful efficiency optimization and compliance with EMFs exposure limits, prompting research on optimized core and coil designs to enhance energy transfer effectiveness in 10 kW IPT chargers^31^. Additionally, safety concerns necessitate regulatory compliance, as demonstrated in an 8 kW WPT system evaluated against ICNIRP 2010 exposure limits to ensure safe human exposure levels^32^. The ongoing development of resonant inductive coupling and high-frequency power electronics is expected to enable even higher power levels while maintaining efficiency and safety, playing a crucial role in the widespread adoption of WPT for public transportation and fleet applications^33^. The author in^8^ introduces a design for a magnetic coupler shaped as Double-D (DD) on both sides, optimized for in-motion inductive charging. This charger is capable of transferring 200 kW of power across a 250 mm air gap with an efficiency of 91.88% and operates at a frequency of 85 kHz.

Furthermore, demonstration and pilot projects are crucial for addressing challenges in this technology, identifying solutions, and helping to establish standard practices. Notable examples of such projects include FABRIC and UNPLUGGED. The FABRIC project began in 2014 and concluded at the end of 2017^34^. It has shown the practicality of using DIPT in various scenarios, including high-power applications (20 kW) and the transition from stationary to speeds of up to 100 km/h. The project also investigated factors such as interoperability, electromagnetic compatibility (EMC), and the potential effects of electromagnetic fields (EMFs) on human health in the countries involved^35^. The study concluded that the overall efficiency of the DIPT system is a critical factor in determining its implementation, with an efficiency range of 80–90% expected when the transmitter coil aligns with the vehicle pad. Road construction also plays a significant role in the system’s efficiency and reliability, as placing a coil on the ground requires thorough analysis to prevent field overlap. UNPLUGGED is the second project, which ran from 2012 to 2015. This project evaluated the impact of the IPT system for EVs on users in urban areas and assessed the technology’s potential to extend the driving range^36^. To achieve its objectives, the UNPLUGGED project explored the interoperability, practical challenges, technical feasibility, and the social and economic impacts of inductive charging. It also included an economic design and feasibility study for in-route dynamic charging. The project successfully developed and implemented two inductive chargers: a 3.7 kW charger (tested for efficiency) and a 50 kW charger (designed to accommodate two different vehicles with varying restrictions and conditions, providing full power to both and enhancing flexibility)^37^. For a comprehensive review of WPT technologies surpassing 11 kW, including recent advancements and trends, refer to^4,38^.

Safety is a critical concern in wireless EV charging, as several risks arise from EMFs, power losses, and thermal effects. One major issue is over-temperature risk, particularly in the ground pad of wireless charging stations. Research shows that the surface of the ground pad can overheat due to inefficient power transfer, leading to thermal degradation and potential fire hazards. In^39^, wireless EV charging technology addresses range anxiety but raises thermal safety concerns. Power losses cause temperature increases on the ground pad surface, potentially leading to skin injuries or ignition of foreign objects. Misalignments between the ground pad and vehicle pad worsen temperature rise, reducing efficiency. Using a 6.6 kW commercial system, the study calculates overtemperature-related losses and conducts FEM simulations. The results show that misalignments, such as horizontal displacement over 98.86 mm or angular offset beyond 9.91°, pose severe over-temperature risks, emphasizing the need for safety evaluations based on IEC standards. Wireless chargers generate heat primarily due to electromagnetic losses, which are worsened by misalignment between the charging coils. Such misalignment reduces power transfer efficiency, increasing resistive losses and eddy currents, which create localized hotspots and lead to over-temperature conditions in both the charging pad and nearby infrastructure^40^. In^41^, a dynamic study on the thermal risks of wireless EV chargers found that heat dissipation and power losses are strongly affected by the spatial alignment of the transmitter and receiver coils. Misalignment reduces coupling efficiency, causing higher leakage inductance and increased Joule heating. This not only raises the temperature of the coils but also disrupts the thermal stability of surrounding components, potentially jeopardizing the system’s reliability and safety. Electromagnetic radiation from wireless chargers can interfere with nearby electronic devices and pose potential health risks due to prolonged exposure, with safety evaluations indicating that high-intensity magnetic fields in compact wireless charging stations may adversely affect human health, particularly in close proximity to the charging system^42^. Additionally, high-power wireless chargers exceeding 20-kW significantly increase fire risks due to excessive heating of metal components near the ground-side coil, while insufficient thermal regulation can trigger thermal runaway in adjacent components, further escalating the likelihood of electrical fires^43^. To mitigate these risks, improved shielding techniques are essential to reduce electromagnetic emissions and heat concentration, alongside enhanced cooling systems to regulate temperature and prevent overheating in the charging assembly. Moreover, better coil alignment mechanisms can optimize power transfer efficiency and minimize power losses, ultimately improving system safety. Ensuring thermal stability and electromagnetic safety in wireless EV charging is crucial for preventing overheating, reducing fire hazards, and maintaining user safety, necessitating further advancements in shielding technologies, dynamic alignment, and cooling mechanisms to enhance the reliability and sustainability of these systems. The authors in^44^ examines the exposure limits of EMFs, safety issues, and shielding methods in wireless EV charging (WEVC) systems. EMFs emissions from wireless power transfer may disrupt vehicle electronics and pose health risks. Regulatory organizations have established exposure limits, and various shielding techniques, both passive and active, are explored. Tests conducted at Oak Ridge National Laboratory (ORNL) demonstrate the effectiveness of shielding in high-power applications. The study^45^ presents the alternating voltage phase coil (AVPC), a novel solution to reduce electromagnetic interference (EMI) and EMFs leakage in EV wireless charging systems. The AVPC, incorporating sequential inversion winding (SIW), reduces electric field (*E*) emissions by up to 85% compared to traditional coils, while maintaining power transfer efficiency. It complies with ICNIRP and European Directive EMF exposure limits, making it a viable solution for EV wireless charging. In^46^, the study explores electromagnetic compatibility (EMC) in wireless power transfer (WPT) systems for EV charging, focusing on shielding technologies to reduce electromagnetic interference (EMI). It reviews both active and passive shielding methods and evaluates materials like ferromagnetic substances, conductive metals, and composites. The paper stresses the importance of adhering to international safety standards, such as ICNIRP, to minimize human exposure to EMFs. Through experimental and simulation studies, the effectiveness of these shielding techniques is assessed, and optimization strategies for balancing shielding efficiency with WPT system performance are discussed.

WEVC has gained significant attention due to its convenience and potential for widespread adoption; however, the safety of EMFs generated by these systems remains a critical concern. EMFs pose risks such as human exposure, EMI, and thermal effects, necessitating compliance with international safety standards. The primary concern regarding EMFs in WEVC systems is their potential health impact, with research indicating that exposure to intermediate-frequency (IF) EMFs must be evaluated against safety limits established by organizations like the ICNIRP and the IEEE^44^, as studies define safe distances for pedestrians and vehicle occupants due to potential risks from prolonged exposure to high-intensity near-field magnetic fields^47^. Compliance with EMFs safety guidelines is crucial, with SAE J2954 outlining criteria for EMC and EMFs safety, ensuring exposure remains within recommended levels^48^, while the specific absorption rate (SAR) is highlighted as a main parameter for evaluating human exposure levels^49^. To mitigate EMFs exposure, various shielding techniques have been developed, such as metal shielding boards and optimized coil designs, which significantly weaken magnetic field strength and reduce exposure risks while maintaining power transfer efficiency^50,51^. Beyond EMFs exposure, thermal effects are another safety concern, as high-power WEVC systems generate heat due to electromagnetic losses, which can lead to overheating and fire hazards if not properly managed^52^. Effective thermal regulation and cooling mechanisms are essential to prevent component degradation and ensure operational safety. Thus, ensuring the safety of EMFs in wireless EV charging requires a multi-faceted approach, including adherence to international safety standards, shielding technologies, optimized system design, and thermal management strategies. While current studies have defined safety guidelines, ongoing research is necessary to enhance system efficiency and minimize potential risks.

Thermal risks in Foreign Object Detection (FOD) for Wireless Power Transfer (WPT) are a critical safety concern, particularly in applications such as wireless electric vehicle (EV) charging. Inductive power transfer systems generate strong electromagnetic (EM) fields that can induce eddy currents in unintended metallic foreign objects (FOs), such as screws or beverage cans, leading to significant heating due to eddy currents and hysteresis losses. Studies have shown that such objects can reach dangerously high temperatures, potentially causing burns, fires, or system malfunctions if not properly detected and mitigated^53^. A particularly complex challenge in WPT systems is the presence of noncooperative metal objects, which do not actively participate in power transfer but still absorb energy from EM fields. Recent research has focused on enhancing detection techniques to minimize these risks, with one approach utilizing pole-to-pole EM distribution characteristics in wireless EV chargers employing double-D (DD) coils to improve metal object detection, enabling more precise localization of foreign objects and mitigating thermal hazards^54^. Various detection techniques and mitigation strategies have been explored, including impedance-based detection, where changes in coil impedance indicate the presence of a metallic object, triggering safety mechanisms^55^; thermal imaging and infrared cameras, which provide real-time monitoring of heat signatures to enable early detection of overheating objects^56^; and the use of multi-polar detection coils, a novel approach that ensures blind-zone-free metal object detection by utilizing strip multi-polar detection coils, significantly enhancing sensitivity and precision^57^.

This study evaluates the safety aspects of the two main IPT system configurations. It examines the safety level associated with an 11.1 kVA power rating for both CP and DDP configurations implemented on the ground side, as well as the CP and DDP architectures employed on the vehicle side. The main contributions of this research include:

- Constructing and modeling 3D finite-element designs in accordance with the guidelines suggested in SAE j2954 (power level of WPT3 (11.1 kVA) and ground clearance of Z3-class requirements) for:
  - A circular pad as universal transmitter (CPT).
  - A DD pad as universal transmitter (DDPT).
  - A circular pad as receiver (CPR).
  - A DD pad as receiver (DDPR).
- Designed MATLAB Simulink circuits for each configuration to determine optimal operating frequencies (79–90 kHz), resonant network components, and filter parameters for maximum efficiency (up to 95.93%) and rated power transfer.
- Evaluated electromagnetic field (EMFs) and electric field (*E*) emissions under ideal alignment and misalignment conditions (lateral, angular) for the proposed charging systems (DDPT-DDPR, DDPT-CPR, CPT-CPR, and CPT-DDPR). Confirmed compliance with ICNIRP 2010 guidelines (B ≤ 15 µT, E ≤ 83 V/m) for all configurations.
- Demonstrated that CPT-CPR and DDPT-DDPR configurations met stricter 1998 ICNIRP limits (B ≤ 6.25 µT), while DDPT-CPR and CPT-DDPR required a 6–9% power reduction to comply.
- Quantified EMFs performance under worst-case misalignments (ΔX =  ± 75 mm, ΔY =  ± 100 mm, angular deviations up to 10°), showing fields remained within safety thresholds for all tested scenarios.
- Verified that both Circular Pad (CP) and Double-D Pad (DDP) receivers’ function effectively with universal transmitters (CPT or DDPT), ensuring compatibility across configurations.
- Integrated ferrite cores and aluminum shielding to minimize stray EMFs, achieving high efficiency (up to 95.93%) while maintaining safety.
- Confirmed all configurations adhered to electric field limits (E ≤ 83 V/m) under all test conditions, ensuring safety for humans and medical devices.

The remainder of the manuscript is structured as follows: The detailed steps for modeling the four proposed magnetic designs are discusses in “Coupler architecture modeling”, then the compensation networks are presented in “Resonant networks (compensation circuits)”. EMFs assessment is conducted in “Leakage electromagnetic fields (EMFs)”, in addition to an analysis of the circuit models is conducted in cases of ideal alignment and misalignment circumstances to get the maximum values of EMFs. In “Results and discussion”, the findings of both the magnetic field and the electric field are discussed and compared. Finally, the outcomes are summarized in “Conclusion and future work”.

## Coupler architecture modeling

The complex design of the inductive pad makes it difficult to obtain an exact analytical solution for the distribution of electromagnetic fields (EMFs). To analyze the EMF, specialized numerical techniques are used. Ansys Maxwell Software provides various methods, including the magneto-static approach, which allows for the extraction of magnetic coupler parameters and the determination of EMF values. 3D finite-element models (3D FEMs) are created and assessed following the guidelines of the J2954 standard, which address factors such as transferred power levels, coil and shielding dimensions (magnetic and metallic), air gap distances, and performance criteria^28^.

### Air gaps and power requirements

It is necessary to model the charging coupler such that it provides the EV battery with enough power over the appropriate period of time. There are additional limitations when producing an inductive charger for electric cars because of this necessity. The charger’s operating power level and the magnetic coupler’s installation site’s ground clearance are the two primary restrictions. The J2954 standard specifies four power levels that are appropriate for light-duty electric car inductive charging. In this range of power transmission, levels span from 3.7 kVA to 22 kVA. WPT1 represents the initial level, delivering a power output of 3.7 kVA. WPT2 expresses the 2nd level, which transfers power of 7.7 kVA. WPT3 and WPT4 represent the 3rd and 4th levels, respectively, which transmit power of 11.1 kVA and 22 kVA. Each power level corresponds to one of three categories of ground clearance, ensuring compatibility with various makes and models of light-duty electric vehicles.

The distance measured perpendicularly from the surface of the ground to the undermost of the vehicle-attached pad is known as the ground clearance. The vertical span encompassed by these three categories varies between 100 and 250 mm. The initial category, referred to as the Z1-class, spans from 100 to 150 mm. The 2nd category, denoted as the Z2-class, encompasses heights ranging from 140 to 210 mm, while the 3rd category, termed the Z3-class, spans from 170 to 250 mm. The vertical space separating the lower surface of the receiver coil from the upper surface of the transmitter coil is referred to as the actual vertical magnetic distance, commonly termed as the air gap. Three mounting options exist for placing the transmitter pad on the ground side. While installing it below ground level (with an air gap distance greater than ground clearance) provides protection against damage and vandalism, this setup poses challenges for accessing the transmitter pad for maintenance or repairs. In the second installation scenario, the transmitter pad is elevated above the ground (when the air gap distance is less than the ground clearance), facilitating easier installation, maintenance, and repair procedures. Conversely, the last installation method for the transmitter pad involves positioning it flush with the ground, providing a level surface that enables maintenance and repair while also offering protection against damage and vandalism. Hence, this investigation opted for the second installation approach, wherein the transmitter is positioned above ground level, indicating that the air gap distance is smaller than the ground clearance. Additionally, this paper operates within the 3rd power level, corresponding to 11.1 kVA with the maximum air distance (Z3-class), with all analyses conducted at the minimum Z3-class height of 170 mm. Two distinct pads, DDP and CP are employed as universal transmitters, with corresponding options for receiving pads: DDP and CP. Consequently, the analysis is conducted on four configurations, namely DDPT-DDPR, DDPT-CPR, CPT-CPR, and CPT-DDPR to assess the safety implications of these charging systems for organisms and pacemakers.

### Modeling of DDPT and CPT for 11.1-kVA

**Fig. 2:**
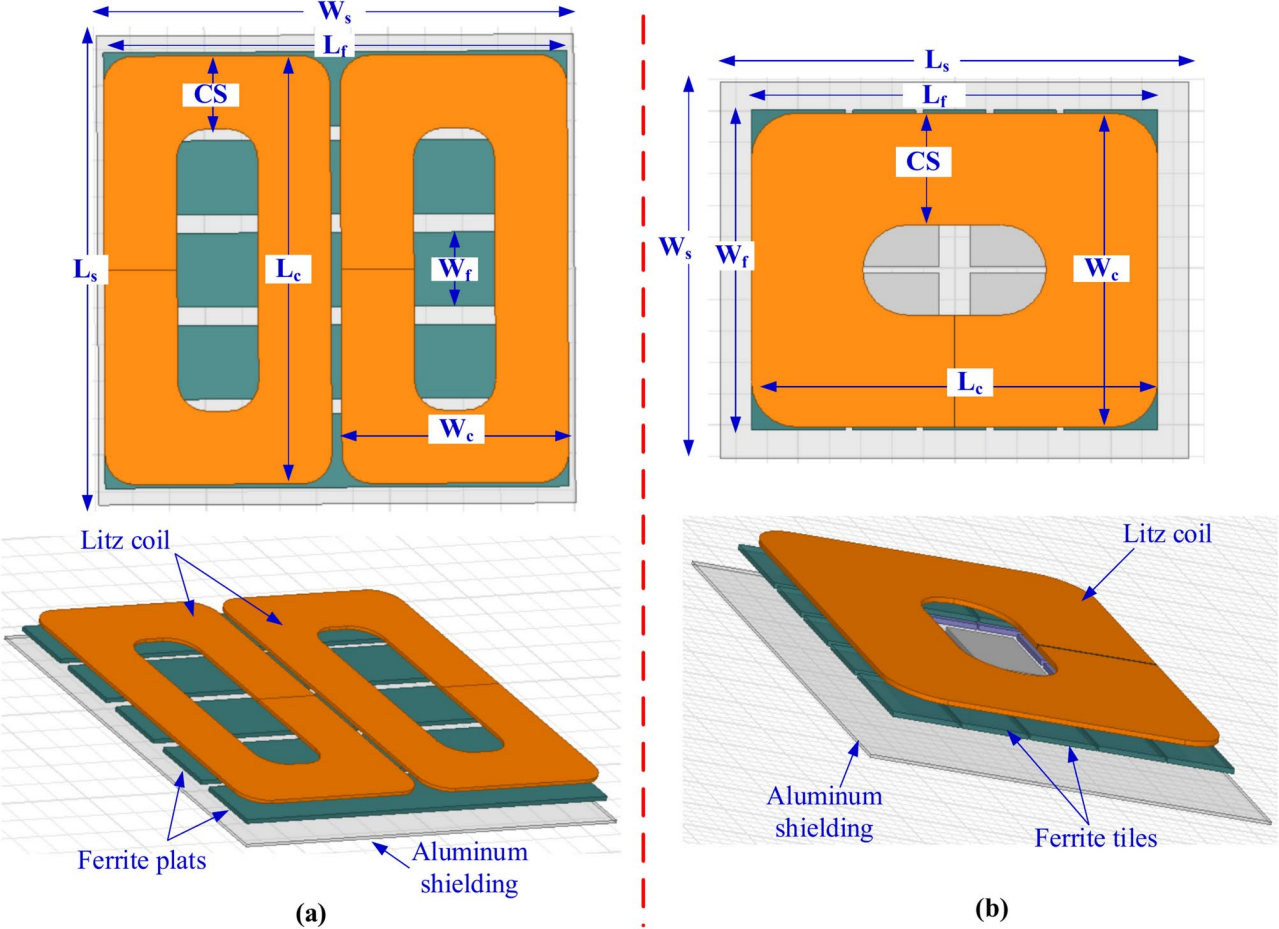
Magnetic modeling of the transmitters for power level of 11.1-kVA: (**a**) DDPT, and (**b**) CPT.

To model the Double-D Pad Transmitter (DDPT) and Circular Pad Transmitter (CPT), 3D Finite Element Models (FEMs) are developed in accordance with the specifications and guidelines set by the SAE J2954 standard. The DDPT comprises two identical single-layer coils arranged in a D-shaped configuration. As shown in Fig. 2a, the coil windings of the DDPT are constructed using litz wire with a 5 mm diameter to facilitate current flow. These coil windings are represented as unified block models, allowing for flexibility in adjusting the number of turns within the model as required. Using blocked turns for coils is a widely accepted approach in various 3D FEM-based applications, including inductive charging systems. This method does not impact the accuracy of results but significantly reduces computational complexity and processing time, thereby enabling efficient analysis.

The DDPT pad incorporates five elongated cores that act as field collectors. These cores are made from ferrite N87 material, known for its high permeability and low conductivity, which enhances performance at high frequencies while minimizing losses and reducing eddy currents. Each core is horizontally spaced 20 mm apart, with a thickness of 6 mm and positioned 1 mm below the coil windings. Additionally, an electrically conductive aluminum plate is placed beneath the ferrite layers, maintaining a separation of 1.3 mm. This plate, with a thickness of 4 mm, plays a crucial role in mitigating stray electromagnetic fields (EMFs)^58^.

On the other hand, adhering to the standards outlined in the SAE J2954 standard, a model for a CPT was constructed. It comprises a two-layer coil constructed from litz wire with a 5 mm diameter, and Fig. 2b illustrates the arrangement of its 16 turns represented as a unified copper block. Additionally, it includes three ferrite layers each with a thickness of 5 mm. These layers are composed of numerous small plates, or tiles, measuring 100 × 100 × 5 mm^3^ and 100 × 150 × 5 mm^3^. An aluminum plate, 3 mm thick, is suspended beneath the ferrite tiles at a distance of 15 mm. This plate functions as a passive shield to diminish the stray field. Table 1 provides the dimensions and specifications of the 3D FEMs for both DDPT and CPT.

**Table 1:** Dimensions of DDPT and CPT at power level of 11.1-kVA.

Para	No. of turns	*L*_*c*_ (mm)	*L*_*f*_ (mm)	*L*_*s*_ (mm)	*W*_*c*_ (mm)	*W*_*f*_ (mm)	*W*_*s*_ (mm)	*CS* (coil side) (mm)
DDPT	6	580	630	635	308	100	648	99
CPT	16	650	650	750	500	510	600	178.1

### Modeling of DDPR and CPR for 11.1-kVA

**Fig. 3:**
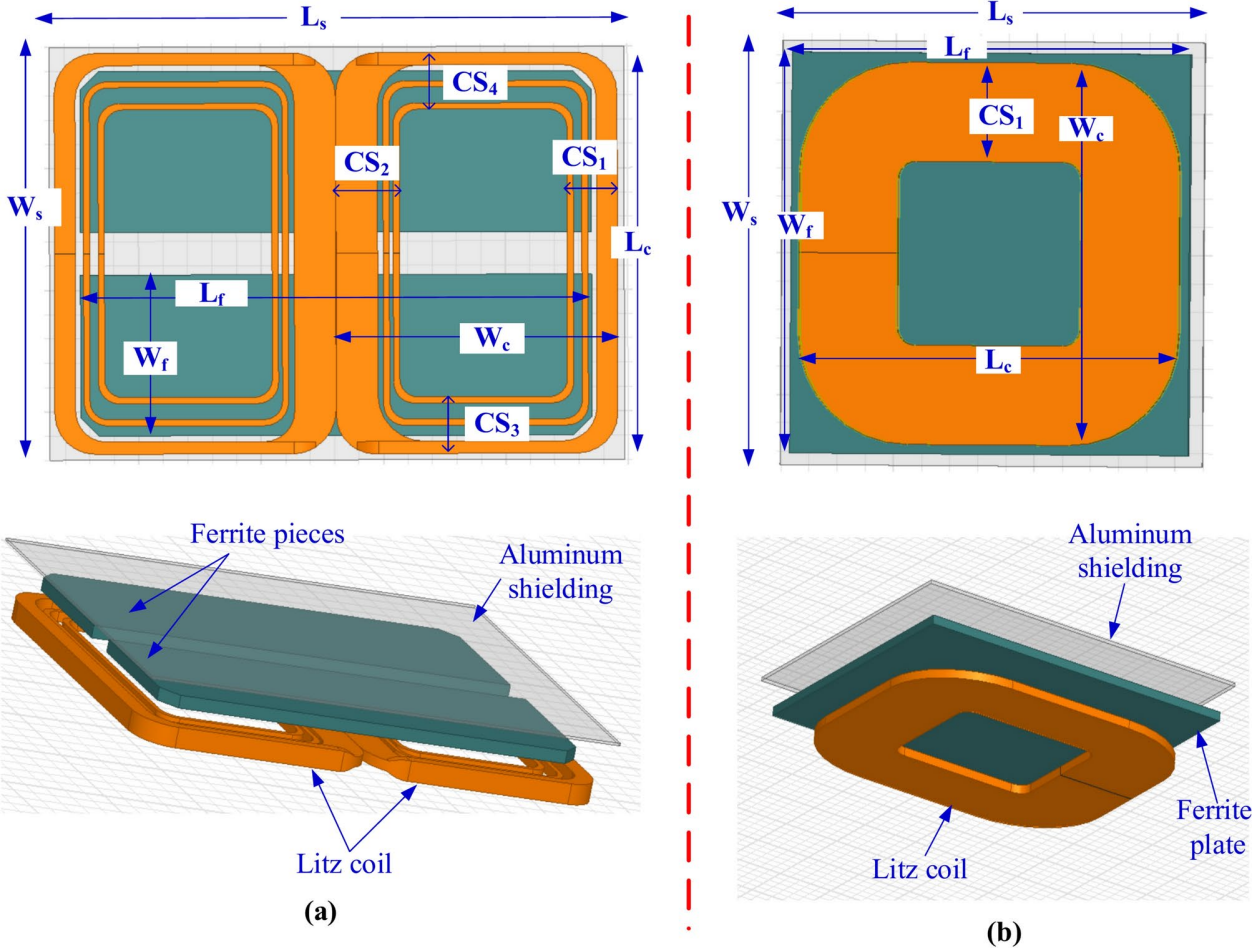
Magnetic modeling of the receivers for power level of 11.1-kVA and Z3-class: (**a**) DDPT, and (**b**) CPT.

EVs equipped with DDPR or CPR configurations must be capable of charging their batteries when using a DDPT ground pad. If the DDPT is replaced with a CPT, the charging performance remains unchanged. To ensure this functionality, the 11.1-kVA DDPR and CPR configurations are simulated in accordance with the SAE J2954 guidelines. The DDPR consists of two identical D-shaped components with nonlinear spacing between them, along with two strata coils made from 5-mm-diameter litz wire. The winding structure of the DDPR is designed so that the litz wires are aligned side by side within the same plane on the middle coil side, while they are stacked on top of each other on the outer coil sides. As shown in Fig. 3a, each D-shaped coil has three turns to replicate this configuration. The DDPR coil is specifically designed with reduced outer side dimensions to minimize stray magnetic fields, whereas the middle coil side is wider to concentrate the magnetic field in that region. Positioned above the receiver coil, the ferrite material consists of two cores, each measuring 418 mm in length and 10 mm in thickness. These cores are placed 1.6 mm from the litz wire turns and are horizontally spaced 35 mm apart. Additionally, a 2 mm thick aluminum plate is positioned above the ferrite cores, maintaining a 0.4 mm gap between them, serving as passive shielding to mitigate electromagnetic interference.

The CPR configuration is designed to be compatible with EV charging when using either DDPT or CPT as the transmitter. It features a blocked-turn coil made of litz wire, with each wire having a 5 mm diameter. Positioned 1 mm above the coil turns is a 5-mm-thick ferrite plate, as illustrated in Fig. 3b. To enhance performance and reduce electromagnetic interference, passive shielding in the form of a 2 mm thick aluminum plate is incorporated, placed 5.6 mm above the ferrite layer. The receiver’s dimensions are dependent on the Z-class of the target vehicle, with larger receiver coils required for greater air gaps. Table 2 provides detailed specifications for both the DDPR and CPR configurations.

**Table 2:** Dimensions of DDPR and CPR at WPT3 power level for Z3-class.

Para	DDPR	CPR
No of Turns	3 (twisted pair)	8
Airgap	170-mm	170-mm
*L*_*c*_	330-mm	380-mm
*L*_*f*_	418-mm	400-mm
*L*_*s*_	470-mm	420-mm
*W*_*c*_	230-mm	380-mm
*W*_*f*_	132.5-mm	400.5-mm
*W*_*s*_	390-mm	420-mm
*CS*_*1*_	41-mm	99.99-mm
*CS*_*2*_	53.18-mm	
*CS*_*3*_ = *CS*_*4*_	46.5-mm	

### Evaluation of magnetic parameters

**Fig. 4:**
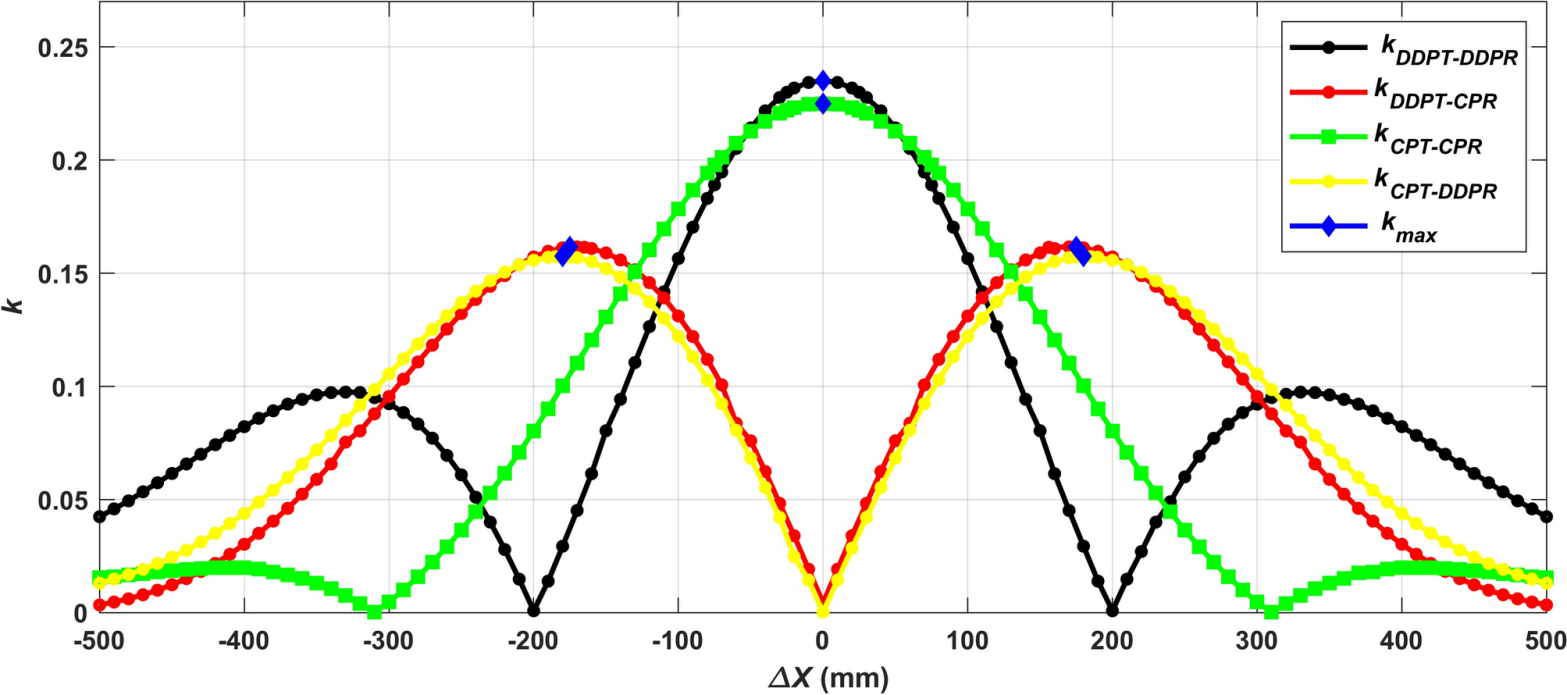
Locations of the maximum values ​​of *k* for the four various models at 11.1kVA along driving direction.

Two analyses were carried out on the DDPT when it was used as a global transmitter at the ground side: One involving the DDPR and the other involving the CPR on the car side. An extra examination was carried out employing both pad variants (DDPR and CPR) on the car side, with the CPT serving as the universal transmitter. In both scenarios, the magnetic parameters for the four designs (DDPT-DDPR, DDPT-CPR, CPT-CPR, and CPT-DDPR) were determined. These parameters include the self-inductance of the transmitter and receiver coils (*L*_*1*_, *L*_*2*_), and the coupling factor between them (*k*). *L*_*1*_, *L*_*2*_ and *k* were obtained under diverse conditions, with priority given to achieving the highest coupling factor k, at the minimum of the air gap distance for Z3-class (170 mm) and installing the pad above the horizontal plane of the ground, thereby facilitating both installation and maintenance of the ground side pad. The coupling factor (*k*) was examined concerning the driving direction {misalignments in X-axis direction (*∆X*)}. Across all designs, the correlation between *k* and the variation in ∆X was graphed from -500 mm to 500 mm to identify positions where *k* is at its maximum, as depicted in Fig. 4.

As shown, the highest *k* values for the DDPT-DDPR and CPT-CPR models occur when their centers are aligned (ideal alignment), whereas for the DDPT-CPR and CPT-DDPR models, the highest *k* values are achieved when there is an offset between the ground coil and car coil.

**Fig. 5:**
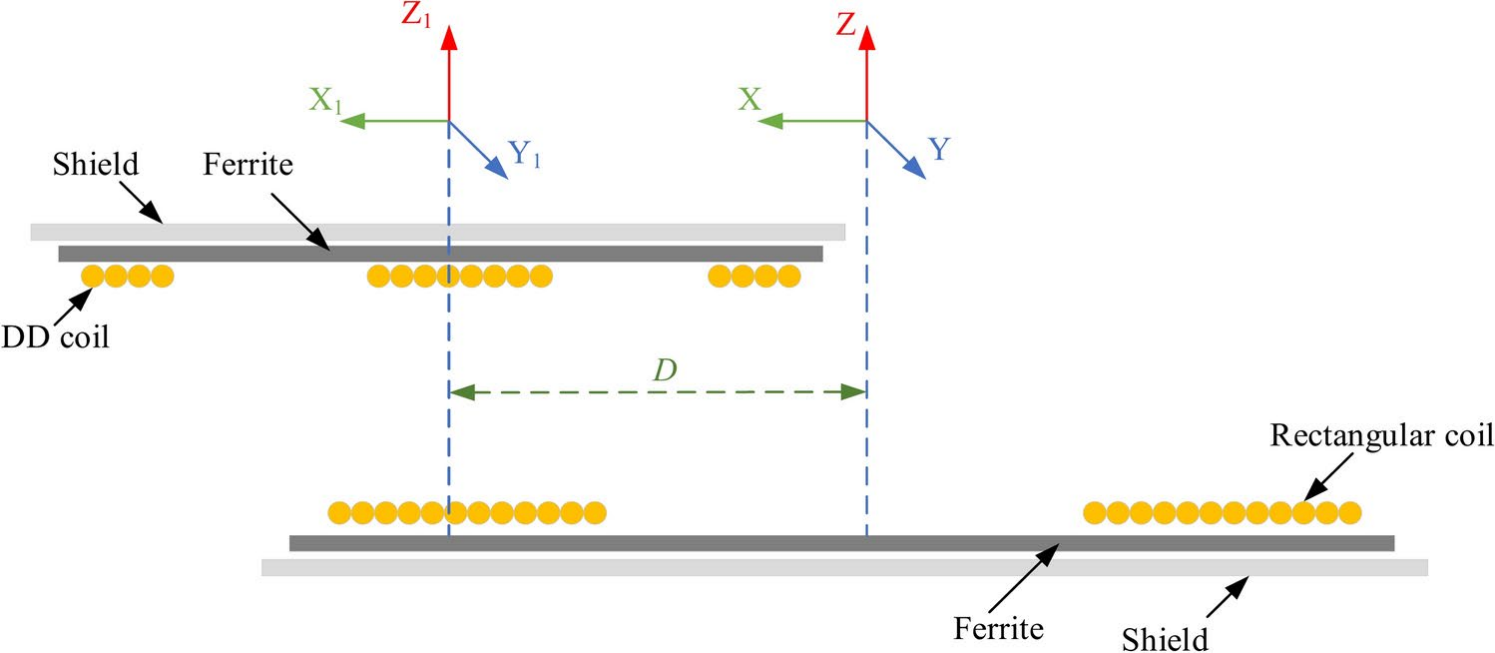
Illustration depicting the inherent displacement between DDP and CP.

As depicted in Fig. 5, the *k* attains zero when the DDPT-CPR and CPT-DDPR models are perfectly aligned. It subsequently increases gradually in both directions until it reaches its peak value when the coil side of the CPT aligns with the middle of the DDPR coil. Consequently, it was found that there exists a natural displacement to achieve the maximum k when utilizing DDP with CP. In the DDPT-DDPR and CPT-CPR models, the location of the maximum k is denoted by the primary coordinate system (X, Y, Z). In case of the DDPT-CPR and CPT-DDPR models, the position of the maximum k is indicated within the new coordinates (X_1_, Y_1_, Z_1_). The distance "*D*" between the two coordinate systems varies depending on the kind of receiving coil. The variations in dimensions, as shown in Table 3, cause this distance to vary for every Z-class.

**Table 3:** Locations of the maximum values of *k* for the four various models.

System configuration	DDPT/DDPR	CPT/CPR	DDPT/CPR	CPT/DDR
*D*	0	0	± 175-mm	± 180-mm
*k*_max_	0.23503	0.22489	0.1616	0.15753

The results derived from examining the four designs were juxtaposed with the benchmarks outlined in the SAE J2954 standard, as presented in Table 4. Upon reviewing the results, it is observed that the values of *L*_*1*_, *L*_*2*_, and *k* for all designs remain within the specified limits outlined in the SAE J2954 standard.

**Table 4:** Comparison of *L*_*1*_, *L*_*2*_, and *k* for all models with the given range within the SAE j2954.

	DDPT/DDPR		DDPT/CPR		CPT/CPR		CPT/DDPR	
	Design	J2954 rang	Design	J2954 rang	Design	J2954 rang	Design	SAE J2954
*L*_*1*_* (uH)*	70.60	66.70–70.80	69.50	66.70–70.80	38.14	37.40–38.70	37.99	37.40–38.70
*L*_*2*_* (uH)*	13.39	13.00–13.80	39.44	39.40–40.20	40.17	39.40–40.20	13.47	13.00–13.80
*k*	0.235	0.136–0.328	0.1616	0.087–0.229	0.2248	0.087–0.229	0.15753	0.136–0.328

## Resonant networks (compensation circuits)

In an IPT system, power transfer efficiency can be significantly affected by leakage inductance, particularly when a wide air gap is present. To mitigate this issue and improve transmission efficiency, compensatory circuits are employed. These circuits also supply reactive power to the system, reducing the apparent power demand on the source. Additionally, by ensuring operation at a unity power factor, they enable soft switching in electronic components. One method of implementing compensation circuits in an IPT system involves using a single capacitor, either in parallel or series, on both sides of the coupling system, as seen in basic compensation techniques^59–61^. Alternatively, a hybrid compensation approach can be used, which incorporates a combination of inductors and capacitors^62–65^. The following sections analyze the components of compensation circuits utilized across IPT systems, integrating various compensation techniques as specified in the J2954 standard.

### DDPT-DDPR and DDPT-CPR resonant elements

**Fig. 6:**
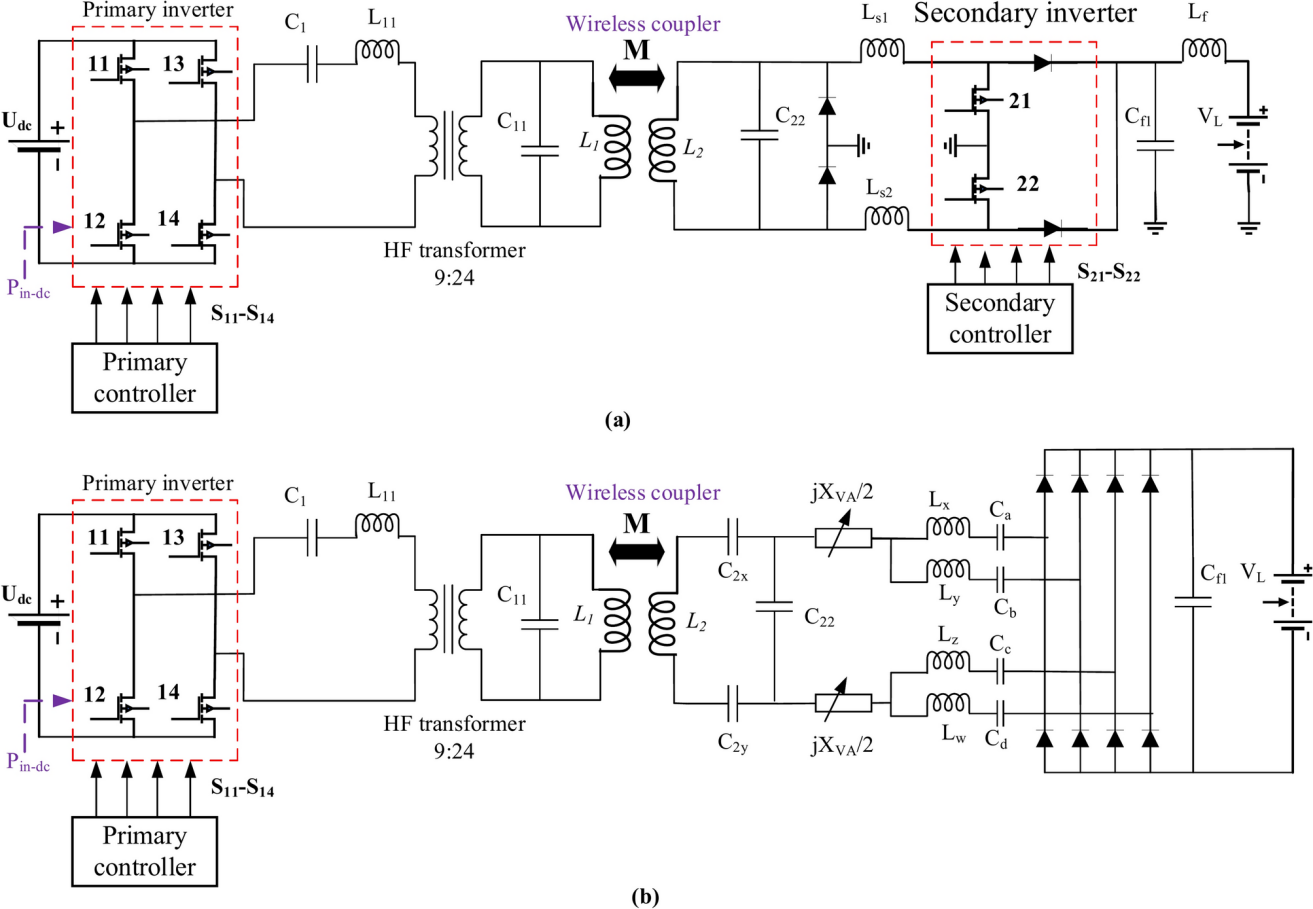
Simulation circuit of WPT3 configurations, (**a**) DDPT/DDPR configuration, and (**b**) DDPT-CPR configuration.

The standard J2954 introduces a resonant network appropriate for DDPT-DDPR coupling system. As depicted in Fig. 6a, this circuit utilizes a hybrid resonance that compromise of an LC and an inductor on both the transmitter and receiver sides. In Fig. 6a, the use of a parallel compensation capacitor (*C*_*22*_) on the secondary side of the wireless power transfer system offers several main advantages, particularly for EV charging applications. The parallel capacitor forms a resonant circuit with the secondary coil (*L*_*2*_), enhancing power transfer efficiency by maximizing resonance at the operating frequency. It also provides load-independent voltage gain, maintaining a stable output voltage despite fluctuations in load conditions. This helps reduce the required VA rating of the secondary-side inverter, minimizing stress on power electronics components. The capacitor improves power transfer capability by boosting the voltage across the rectifier and facilitates better soft-switching conditions, such as zero-voltage switching (ZVS) or zero-current switching (ZCS), which reduces switching losses and enhances overall system efficiency. Additionally, it compensates for reactive power, reducing circulating current in the secondary-side circuit and minimizing conduction losses. Finally, this compensation topology simplifies voltage regulation, making it easier to control the output voltage (*V*_*L*_) across the load. Overall, the use of a parallel capacitor improves efficiency, voltage stability, and power transfer capability, making it an effective solution for wireless EV charging systems. Table 5 contains a list of this system’s electrical specifications. As the sole variable in the DDPT-DDPR system is its operating frequency, MATLAB Simulink was utilized to model the system and identify the suitable frequency of operation. This simulation circuit was analyzed within the frequency spectrum spanning from 79-kHz to 90-kHz, with an incremental step size of 0.5-kHz, consistent with the guidelines outlined in the J2954 standard.

**Table 5:** Electrical requirements of WPT3 DDPT/DDPR configuration.

Para	*C*_*1*_ (nF)	*C*_*11*_ (nF)	*L*_*11*_ (µH)	*L*_*1*_ (µH)	*k*	*L*_*2*_ (µH)	*C*_*2*_ (nF)	*L*_*s1,*_* L*_*s2*_ (µH)	*C*_*f1*_ (µF)	*L*_*f*_ (µH)	*U*_*dc*_ (V)	*U*_*L*_ (V)
DDPT/DDPR	305	50	22	70.60	0.235	13.39	270	250	40	2	800	360

**Fig. 7:**
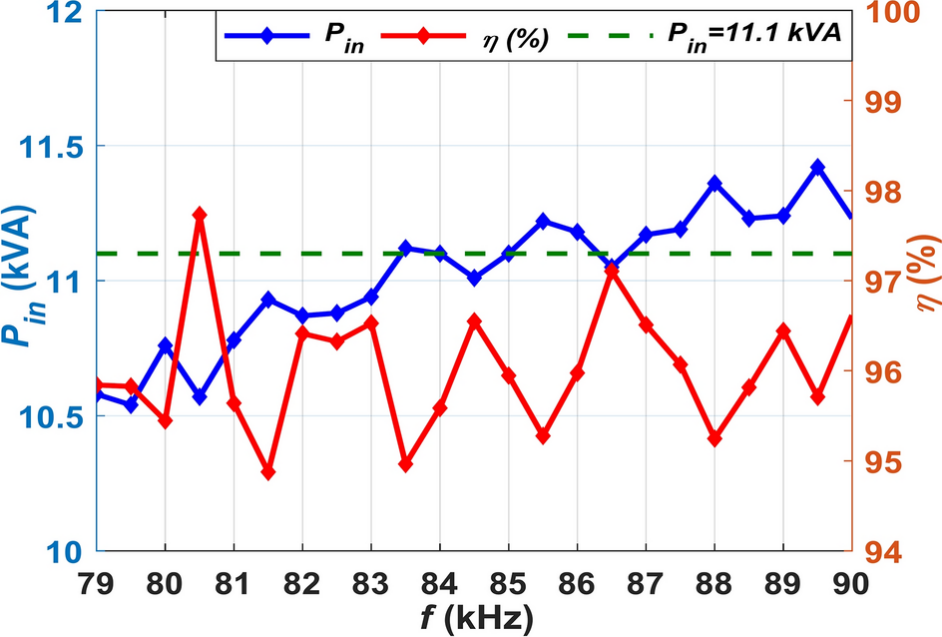
Correlation among *P*_*in*_, *η*, and *f* in DDPT/DDPR.

Figure 7 illustrates the correlation among the frequency of operation (*f*), input power (*P*_*in*_), and DC-DC efficiency (*η*). The frequencies of 85 kHz, 84 kHz, and 83.5 kHz were identified to result in a *P*_*in*_ of 11.1 kVA. Consequently, the system can work at all resulting frequencies; nevertheless, 85 kHz is chosen for the operation of the DDPT-DDPR configuration due to its ability to deliver the maximum output power of 10.652-kW and achieve the maximum efficiency of 95.93%.

Given that the DDPT-CPR system shares the same ground side pad, depicted in Fig. 6b, an LC resonance network is employed on the ground side pad, while LCC compensation technique is employed on the car side pad. The LCC compensation network on the car side pad offers several advantages that enhance the efficiency and performance of wireless EV charging systems. The LCC topology, comprising inductors and capacitors, forms a highly resonant circuit that improves power transfer efficiency while reducing reactive power losses. It ensures load-independent voltage gain, maintaining stable voltage (*V*_*L*_) at the battery side regardless of load variations. The network also achieves improved soft switching, such as Zero Voltage Switching (ZVS) or Zero Current Switching (ZCS), which minimizes switching losses, reduces heat generation, and improves overall system efficiency. Additionally, the LCC topology offers higher tolerance to coil misalignment between the primary and secondary sides, making it especially beneficial for dynamic charging scenarios. The inclusion of inductors reduces circulating currents, minimizing conduction losses and enhancing power handling. The LCC network also provides robust voltage regulation and supports both constant current (CC) and constant voltage (CV) charging modes, addressing various EV battery charging needs. Furthermore, it compensates for reactive power, alleviating the VA burden on power electronics and improving system efficiency. The multi-phase rectifier design, in conjunction with the LCC compensation, results in better DC voltage output with reduced ripple, further enhancing charging performance. Overall, the LCC compensation network significantly improves system efficiency, voltage stability, and tolerance to misalignment, making it a highly effective solution for EV wireless charging applications. Table 6 lists this system’s electrical specifications. The only variables in this simulated circuit are *f* and the car side filter reactance (*X*_*VA*_*/2*). In accordance with J2954, the *f* spans from 79 to 90 kHz, whilst the *X*_*VA*_*/2 *ranges from – 15-Ω to 0-Ω. This implies that the filter exhibits capacitive characteristics and can be symbolized by a capacitor (*C*_*VA*_) with a value ranging from 0.135-µF to 0.179-µF, contingent upon the frequency of operation range. In the specified range, the frequency was incremented by steps of 0.5-kHz. At each frequency, the *C*_*VA*_ value was adjusted by 0.0045 µF, ranging from 0.135-µF to 0.179-µF.

**Table 6:** Electrical requirements of WPT3 DDPT/CPR configuration.

Para	*U*_*dc*_ (V)	*C*_*1*_ (nF)	*L*_*11*_ (µH)	*C*_*11*_ (nF)	*L*_*1*_ (µH)	*k*	*L*_*2*_ (µH)	*C*_*2x*_ (nF)	*C*_*2y*_ (nF)	*C*_*22*_ (nF)	*L*_*x*_ (µH)
DDPT/CPR	800	305	22	50	69.50	0.1616	39.43	250	250	134	54

Para	*L*_*y*_ (µH)	*L*_*z*_ (µH)	*L*_*w*_ (µH)	*C*_*a*_ (nF)	*C*_*b*_ (nF)	*C*_*c*_ (nF)	*C*_*d*_ (nF)	*C*_*f1*_ (µF)	*U*_*L*_ (V)	*X*_*VA*_*/2* (Ω)	–
DDPT/CPR	54	54	54	100	48	48	100	30	400	− 15−0	–

**Fig. 8:**
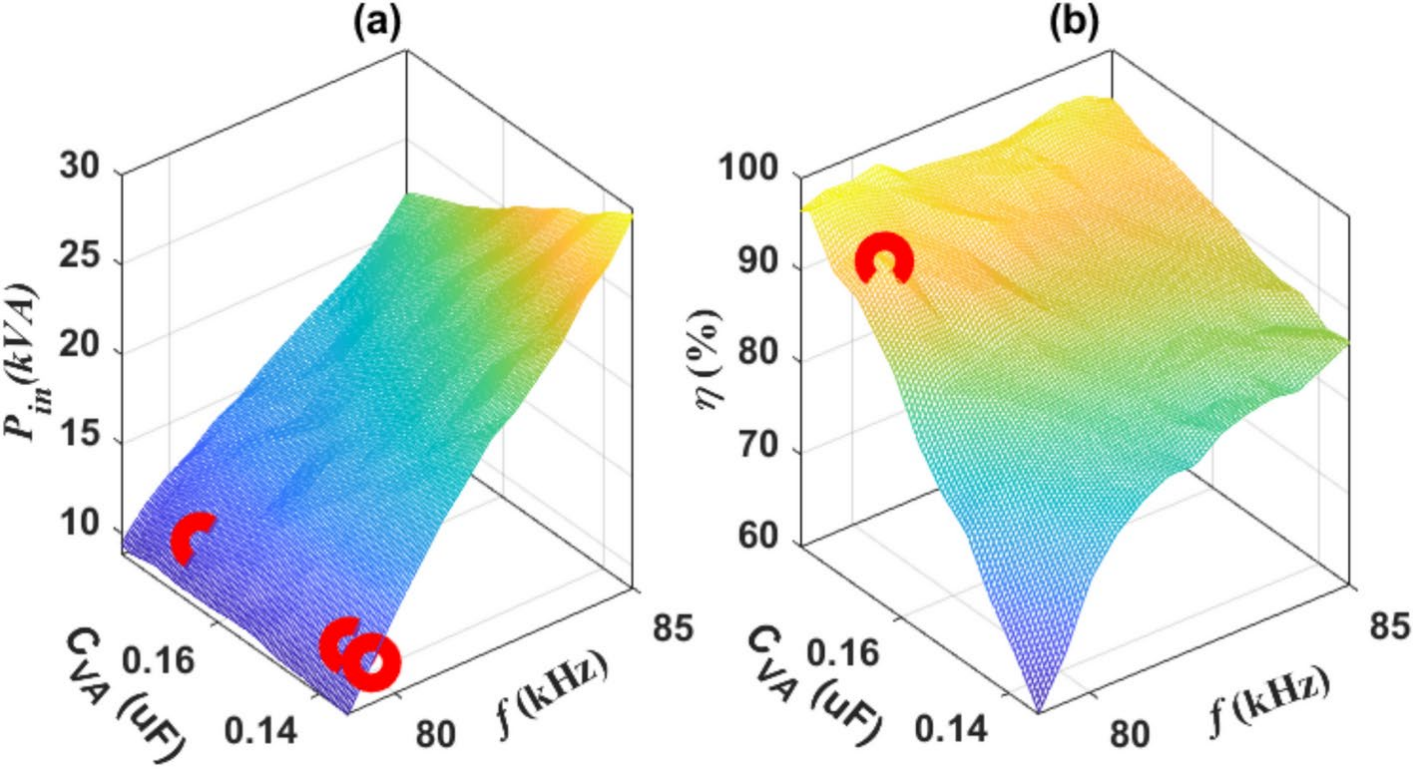
Operational point of DDPT-CPR configuration, (**a**) the correlation among *P*_*in*_, *C*_*VA*_, and* f*, and (**b**) the correlation among *η*, *C*_*VA*_, and* f*.

In Fig. 8a, the correlation among *P*_*in*_, *C*_*VA*_, and *f* is illustrated. It is observed that at a frequency of 79.5 kHz, an input power (*P*_*in*_) of 11.1 kVA can be achieved at various values of *C*_*VA*_. Red circles mark the points corresponding to the *P*_*in*_ of 11.1 kVA. Additionally, it is noted that as the frequency rises, the *P*_*in*_ also increases, leading to a divergence indicative of the operating point where *P*_*in*_ equals 11.1 kVA. Hence, the frequency range from 79 to 85 kHz was deemed sufficient. Given that each identified point yields the same *P*_*in*_, a relation between *f*, *C*_*VA*_, and *η* was established, as illustrated in Fig. 8b, to ascertain the point offering the highest efficiency. The findings indicate that the output power peaks at its maximum (*P*_*o*_ = 10.46 kW), and the efficiency achieves its highest level (*η* = 94.23%) at a frequency of 79.5 kHz and a *C*_*VA*_ of 0.168 µF. A red circle designates the DDT-RR system’s operating point.

### CPT-CPR and CPT-DDPR resonant elements

**Fig. 9:**
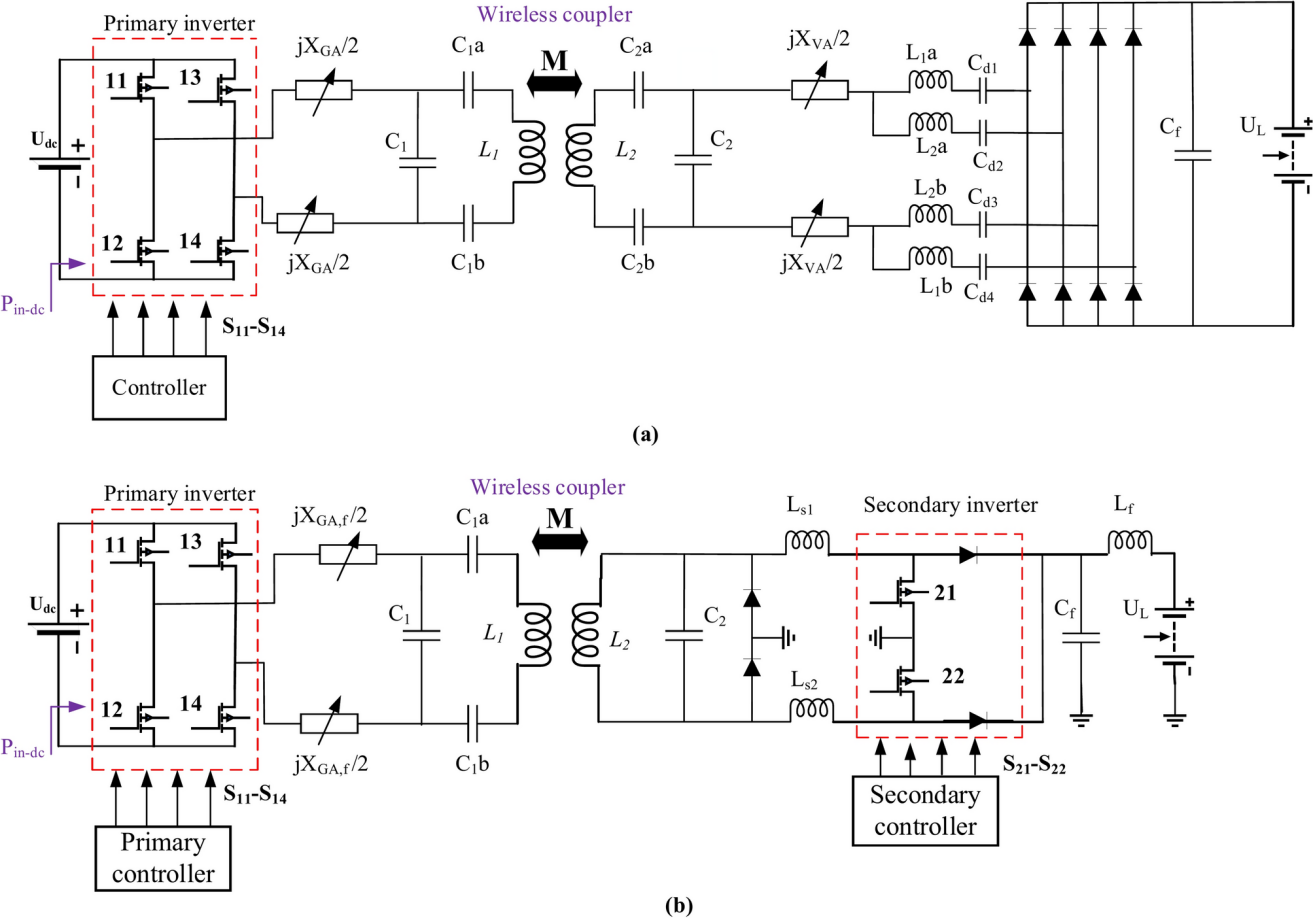
Simulation circuit of WPT3 configurations, (**a**) CPT/CPR configuration, and (**b**) CPT-DDPR configuration.

The CPT-CPR configuration employs the LCCL compensation technique on both the transmitting and receiving sides, as illustrated in Fig. 9a. Table 7 provides the electrical specifications of the CPT-CPR configuration. Except for the given range of the filter reactance at ground side and car side pads (*X*_*GA*_*/2* and *X*_*VA*_*/2*), all electrical values are dictated by the J2954 standard. As *X*_*GA*_*/2* falls within the range of 4–16 Ω, it demonstrates inductive characteristics. This can be represented by an inductor (*L*_*GA*_) ranging from 7.07 µH to 8.05 µH, contingent upon the determined frequency range of 79 kHz to 90 kHz.

**Table 7:** Electrical requirements of WPT3 CPT/CPR configuration.

Para	*U*_*dc*_ (V)	*C*_*1*_ (nF)	*C*_*1a*_ (nF)	*C*_*1b*_(nF)	*C*_*2a*_ (nF)	*C*_*2b*_ (nF)	*L*_*1*_ (µH)	*k*	*L*_*2*_ (µH)	*C*_*2*_ (nF)	*L*_*1a*_ (µH)
CPT-CPR	500	268	307	307	250	250	39.14	0.2248	40.17	134	54

Para	*L*_*1b*_ (µH)	*L*_*2a*_(µH)	*L*_*2b*_ (µH)	*C*_*d1*_ (nF)	*C*_*d2*_ (nF)	*C*_*d3*_ (nF)	*C*_*d4*_ (nF)	*C*_*f*_ (µF)	*U*_*L*_ (V)	*X*_*GA*_*/2* (Ω)	*X*_*VA*_*/2* (Ω)
CPT-CPR	54	54	54	100	48	48	100	30	400	4−16	− 15−0 Ω

A MATLAB Simulink model is developed to simulate the CPT-CPR configuration depicted in Fig. 9a. Analysis of this Simulation model is conducted to ascertain the frequency of operation (*f*), receiver filter capacitance (*C*_*VA*_), and transmitter filter inductance (*L*_*GA*_). This facilitates the transmission of nominal power with the utmost efficiency (*η*), considering the *P*_*in*_ of 11.1 kVA (WPT3) and ideal alignment conditions.

**Fig. 10:**
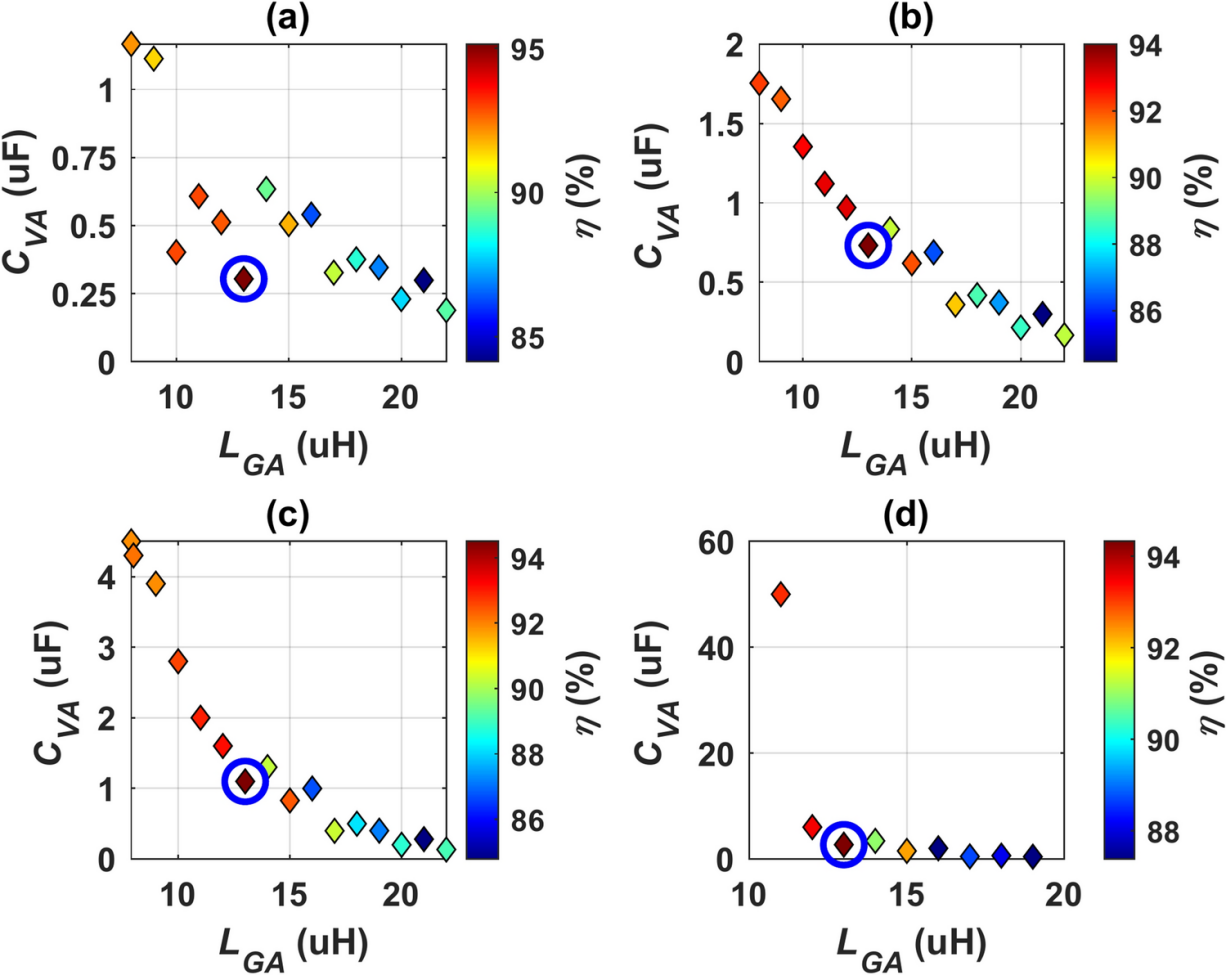
The correlation among, *L*_*GA*_, and *η C*_*VA*_ for CPT/CPR configuration: (**a**) @ *f* equal to 79-kHz, (**b**) @ *f* equal to 79.5-kHz, (**c**) @ *f* equal to 80-kHz, and (**d**) @* f* equal to 80.5-kHz.

Taking into consideration the frequency range specified in J2954, adjustments are made in increments of 0.5 kHz. Figure 10 illustrates the correlation among *η*, *L*_*GA*_, and* C*_*VA*_. It is observed that the input power value deviates further from 11.1 kVA at higher frequencies. Consequently, operating frequency values of 79, 79.5, 80, and 80.5-kHz were identified to meet the required *P*_*in*_. The *L*_*GA*_ values corresponding to each frequency value, were adjusted by one µH, and the *C*_*VA*_ value was fine-tuned at each *L*_*GA*_ until the charging coupler successfully transferred rated power. Subsequently, the *C*_*VA*_ values and the corresponding efficiency values were determined. Table 8 presents the subplots at all frequencies where nominal power transmission is achieved. The operating point is identified by locating the rated power at the maximum *η*. This operating point (*L*_*GA*_ = 13 µH, *η* = 95.04%, *P*_*o*_ = 10.55 kW, *C*_*VA*_ = 0.304 µF, and *f* = 79 kHz) is marked by a blue circle.

**Table 8:** Parameters yielding the maximum *η* at each frequency for the CPT/CPR configuration.

*f*	80.5 (kHz)	80 (kHz)	79.5 (kHz)	79 (kHz)
*L*_*GA*_	13 (µH)	13 (µH)	13 (µH)	13 (µH)
*C*_*VA*_	2.68 (µF)	1.1 (µF)	0.732 (µF)	0.304 (µF)
*η*	94.2 (%)	94.4 (%)	94.1 (%)	95.05 (%)

The resonant circuit of the CPT-DDPR configuration remains consistent due to its utilization of the same CPT. However, on car side, a parallel LC compensation technique is employed, attributed to the utilization of DDP, as illustrated in Fig. 9b. Table 9 exhibits the electrical requirements of the CPT-DDPR configuration. As the same transmitter pad is maintained, this value remains constant once it aligns with the *L*_*GA*_ value determined from the prior analysis. Consequently, the sole variable requiring determination for achieving maximum *P*_*o*_ with optimal *η* is the operating frequency (*f*).

**Table 9:** Electrical requirements of WPT3CPT/DDPR configuration.

Para	*U*_*dc*_ (V)	*C*_*1*_ (nF)	*C*_*1a*_ (nF)	*C*_*1b*_ (nF)	*C*_*2*_ (nF)	*L*_*1*_ (µH)	*k*
CPT-DDPR	500	268	307	307	270	40.166	0.15753

Para	*L*_*2*_ (µH)	*L*_*s1*_ (µH)	*L*_*s2*_ (µH)	*C*_*f*_ (µF)	*L*_*f*_ (µH)	*U*_*L*_ (V)	*X*_*GA*_*/2* (Ω)
CPT-DDPR	13.47	250	250	40	2	320	4–16

**Fig. 11:**
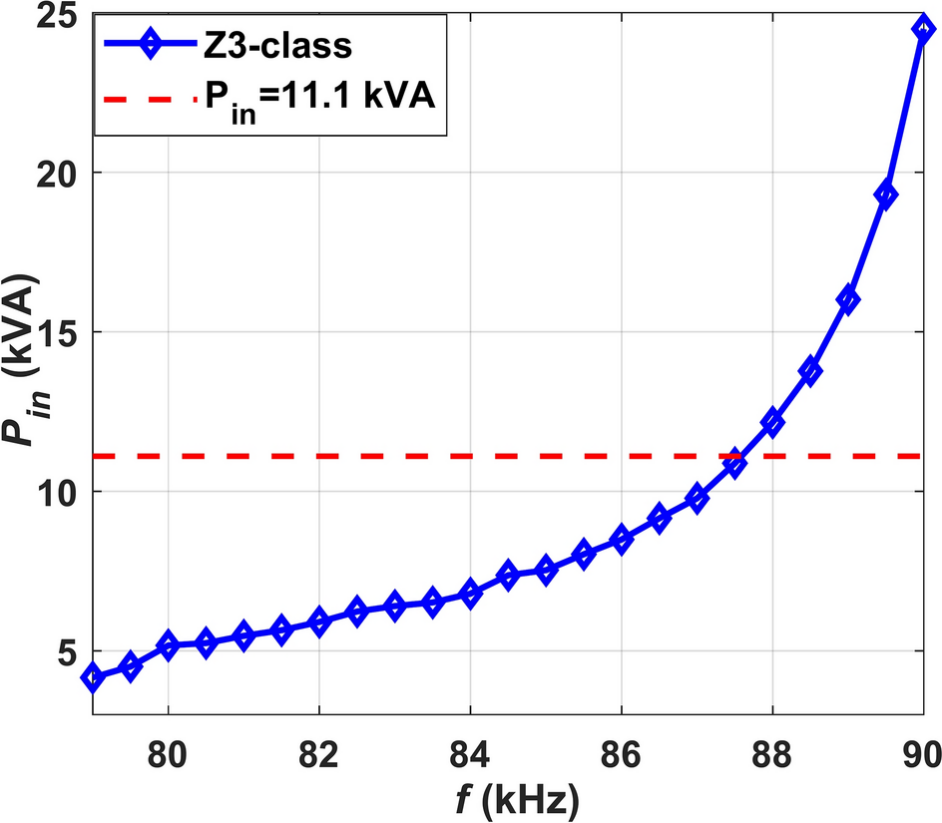
Relationship *f* and *P*_*in*_ of the CPT-DDPR configuration.

MATLAB Simulink is employed to construct the CPT-DDPR configuration and determine the appropriate operating frequency. The *P*_*in*_ and *f* are correlated, as depicted in Fig. 11. The graph illustrates that the *P*_*in*_ of the CPT-DDPR charging system escalates as the *f* rises. One can determine the frequency from the Figure at which the *P*_*in*_ reaches 11.1 kVA. The *η* of the CPT-DDPR configuration is diminished compared to that of the CPT-DDPR configuration, owing to its elevated operating frequency necessity to transfer the equivalent power amount.

## Leakage electromagnetic fields (EMFs)

In a wireless inductive charging system, electromagnetic fields (EMFs) transfer power from the transmitting coil to the receiving coil. A massive amount of electromagnetic fields are produced during the feeding of the transmitter coil, and these fields move across the airgap distance from the transmitting to the receiving side. While some of these fields are lost to the air around the charging system, others intersect with the receiving coil to produce the useful power. If these leaking electromagnetic fields surpass the safe thresholds permitted by international bodies, they present a hazard to the well-being of living organisms in the proximity of the charging system^66^. These fields are capable of generating potent induced currents within human bodies, exposing body tissues to heat stress and endangering people’s health^67^. Furthermore, it interferes with the functionality of portable medical devices, like pacemakers, which has a negative effect on them^68^. Numerous international entities, such as the ICNIRP guidelines, have set forth safety thresholds for electromagnetic field emissions at varying frequencies. In order to define distinct safe limits for electromagnetic fields based on operating frequency, the ICNIRP guidelines were released in two versions, in 1998 and 2010, respectively. The safe limit for the external magnetic field density (*B*) was suggested by the ICNIRP 1998 version to be 6.25 µT^48^, whereas the safe limits for *B* in the ICNIRP 2010 version were 15 µT for pacemakers and 27 µT for living things^69,70^. Following consideration of the ICNIRP guidelines, the SAE j2954 committee determined that 15 µT is the safe general limit for the *B* of the charging system. To be cautious and accommodate both organisms and pacemakers, this value was selected^70,71^. Furthermore, the same guidelines (83 V/m) define the safe limits for electric fields.

**Fig. 12:**
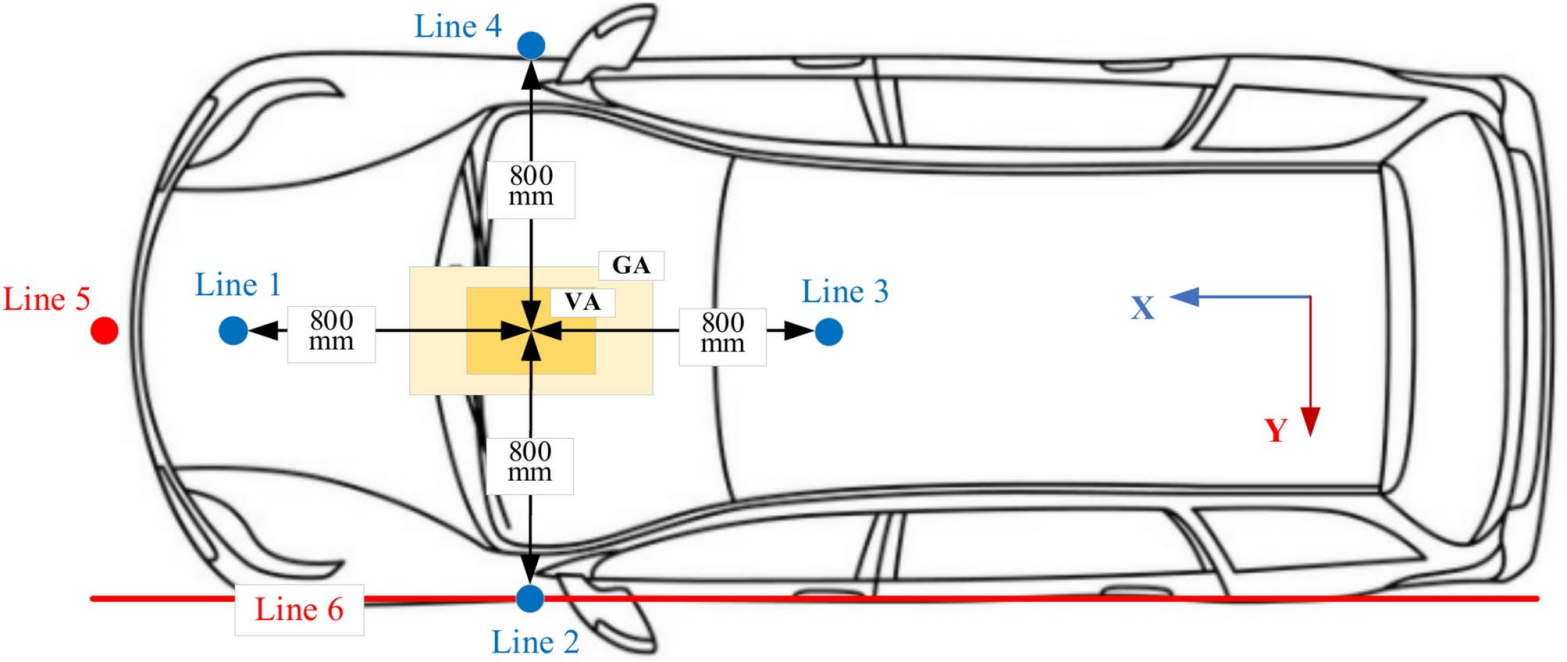
Electromagnetic Fields test points according to the SAE J2954.

The SAE J2954 standard defines two primary methods for measuring electromagnetic fields (EMFs) around the wireless charging system of electric vehicles (EVs): bench setup testing and in-vehicle testing, both designed to ensure compliance with electromagnetic compatibility (EMC) and safety regulations. In the bench setup method, EMFs measurements are taken at four specific directions (north, south, east, and west) relative to the center of the transmitter coil, with a horizontal offset of 800 mm from the coil’s center in each direction to capture the spatial distribution of leakage fields as illustrated in Fig. 12. This setup forms four perpendicular measurement lines extending vertically from ground level through the air gap to a point above the receiving pad, where electromagnetic field intensity is continuously recorded. The front and rear of the EV correspond to perpendicular measurement lines 1 and 3, while the left and right vehicle sides correspond to lines 2 and 4. The most critical measurement occurs at the midpoint between the ground-side charging pad and the vehicle-side receiving pad, as this location represents the strongest coupling region in the inductive power transfer (IPT) system.

The in-vehicle testing method categorizes measurement zones into three distinct regions to assess the impact of EMF exposure on vehicle occupants and external environments. Zone 1 corresponds to the space directly beneath the EV, where emissions are evaluated for potential leakage into nearby ground structures. Zone 2 includes the area around and above the vehicle, covering pedestrian access points and adjacent lanes to ensure EMF emissions remain within permissible exposure limits for bystanders and nearby electronic systems. Zone 3 represents the vehicle’s interior, where exposure levels are assessed for compliance with human safety standards. To systematically validate EMF exposure, three observation lines are established within these zones, forming reference paths along which field strength measurements are conducted. Additionally, specific test points include Line 5, which assesses side exposure outside the vehicle to evaluate EMF emissions affecting adjacent lanes or pedestrians, and Line 6, a roadside exposure test line extending along the vehicle width to analyze electromagnetic interference (EMI) impact on roadside infrastructure or nearby vehicles. These test locations ensure compliance with SAE J2954 standards, maintaining EMF emissions below regulatory thresholds to safeguard passengers, minimize interference with electronic devices, and validate the safety, efficiency, and EMC conformity of EV wireless power transfer (WPT) systems.

The electromagnetic field values are influenced by the arrangement of the transmitter and receiver coils, variations in alignment, and ground clearance. A fundamental characteristic of inductive charging systems is that a vehicle can never be perfectly positioned over the charging coil. Therefore, to realistically simulate real-world conditions, the inductive charging system must account for positional deviations in multiple directions. The SAE j2954 standard states that there are two types of misalignments that the charging system is expected to encounter: angular and linear misalignments. Angular misalignments are represented by the angles Roll (± 2°), Pitch (± 2°), and Yaw (± 10°). Misalignments can occur in the driving direction (ΔX =  ± 75 mm), and/or in the direction of the Y-axis (ΔY =  ± 100 mm), in the direction of the Z-axis (ΔZ). When rotation occurs about the X-axis, this deviation is termed Roll. Meanwhile, if the EV undergoes rotation around the Y-axis, this deviation can be labeled as Pitch. However, the rotational deviation is referred to as Yaw when the EV rotates around the Z-axis^72^.

**Fig. 13:**
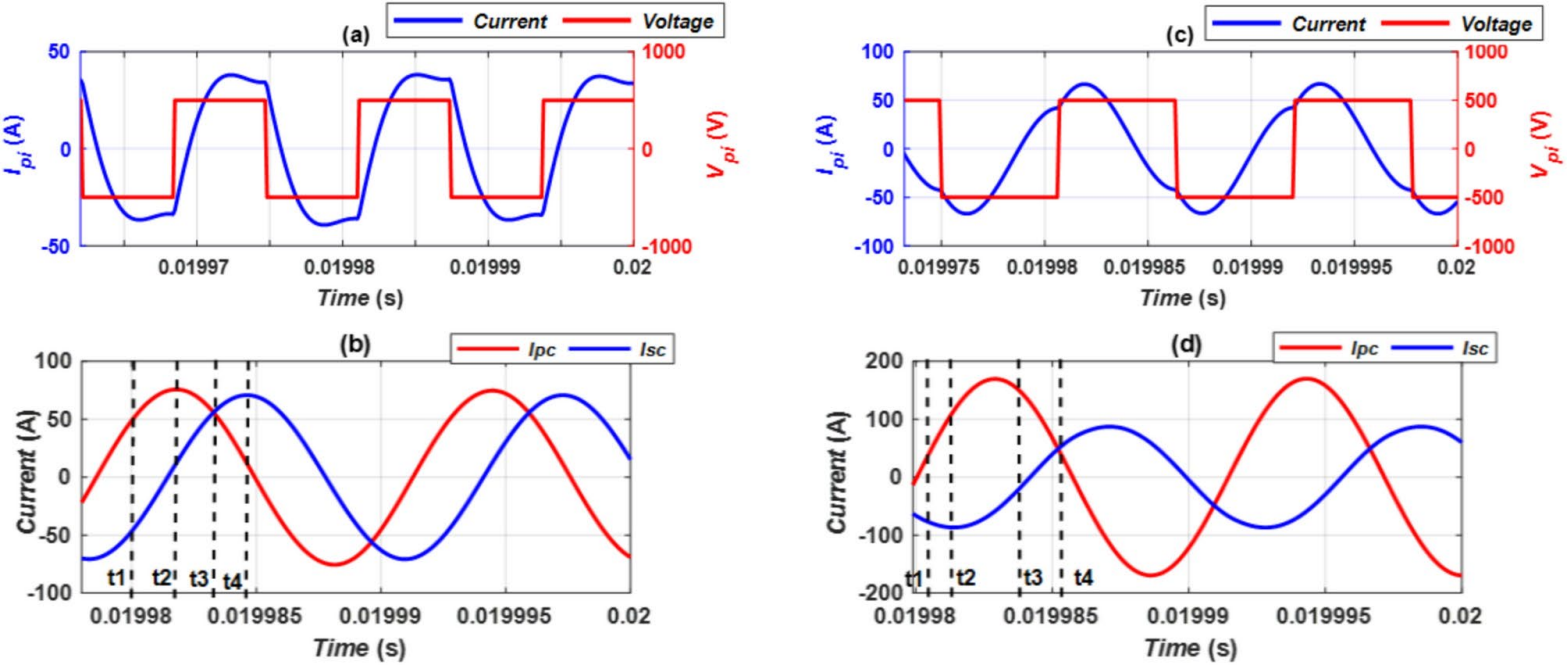
Locations for extracting current in EMF configurations (**a**) *I*_*pi*_ vs. *V*_*pi*_ for CPT-CPR, (**b**) *I*_*pc*_ vs. *I*_*sc*_ for CPT-CPR, (**c**)* I*_*pi*_ vs. *V*_*pi*_ for CPT-DDPR, and (**d**) *I*_*pc*_ vs.* I*_*sc*_ for CPT-DDPR.

An investigation is conducted into the electric circuits of the CPT-CPR and CPT-DDPR configurations. This analysis takes into account the factors associated with optimal alignment conditions. In Fig. 13, output voltage and current for CPT-CPR and CPT/DDPR systems’ primary inverter (*V*_*pi*_, *I*_*pi*_), transmitter coil current (*I*_*Pc*_), and receiver coil current (*I*_*Sc*_) are compared Since coil currents and magnetic fields are primarily related, four instants of current waveforms are confirmed as shown in Fig. 13b,d. (t_1_, t_2_, t_3_, and t_4_). The receiver current (*I*_*Sc*_) and transmitter current (*I*_*Pc*_) are taken out and put into the FEMs at each instant. Taking into account the worst-case misalignment scenario, the magnetic field is evaluated for the CPT-CPR and CPT-DDPR configurations. As both models are alike, the *B* value is assessed at all lines ranging from 1 to 4, and the results are the same for any two opposite lines. Therefore, consideration is given to the findings at lines 1 and 2. Table 10 exhibits the B values for line 1 that located at the front of EV and line 2 which located at one side of EV. For the ongoing analysis, concerning both CPT-CPR and CPT-DDPR configurations, the moment labeled as *t*_*3*_, where the most significant field distributions are observed, is considered.

**Table 10:** Extreme values of magnetic field density at various time points.

Time	CPT-CPR				CPT-DD_R			
	*I*_*Pc*_	*I*_*Sc*_	*B* at Line 1	*B* at Line 2	*I*_*Pc*_ (A)	*I*_*Sc*_ (A)	*B* at Line 1	*B* at Line 2
t_1_	48.74 A	− 46.75 A	1.19 µT	1.14 µT	19.76 A	− 73.12 A	4.26 µT	3.96 µT
t_2_	75.7 A	11.54 A	3.00 µT	3.20 µT	98.03 A	− 84.43 A	6.38 µT	5.94 µT
t_3_	75.83 A	55.95 A	3.01 µT	3.34 µT	158.9 A	− 31.08 A	6.59 µT	7.30 µT
t_4_	18.9 A	70.73 A	1.97 µT	2.04 µT	46.53 A	46.53 A	3.25 µT	3.35 µT

## Results and discussion

Assessing the safety impact of the universal ground-side pads (DDPT and CPT) and various car-side pads (DDPR and CPR) requires analyzing the developed FEMs for the DDPT-DDPR, DDPT-CPR, CPT-CPR, and CPT-DDPR configurations. The safety evaluation, conducted under both linear and angular misalignment conditions, focuses on the most challenging ground clearance scenario (Z3-class). Misalignment assessments for the DDPT-DDPR and CPT-CPR configurations are performed using XYZ coordinates, while the X_1_Y_1_Z_1_ coordinate system is used to account for the natural offset in the DDPT-CPR and CPT-DDPR configurations (as illustrated in Fig. 5). To ensure the safety of the charging system, measurements of electric and magnetic fields were taken in both fully aligned and misaligned states.

### Leakage magnetic field assessment

**Fig. 14:**
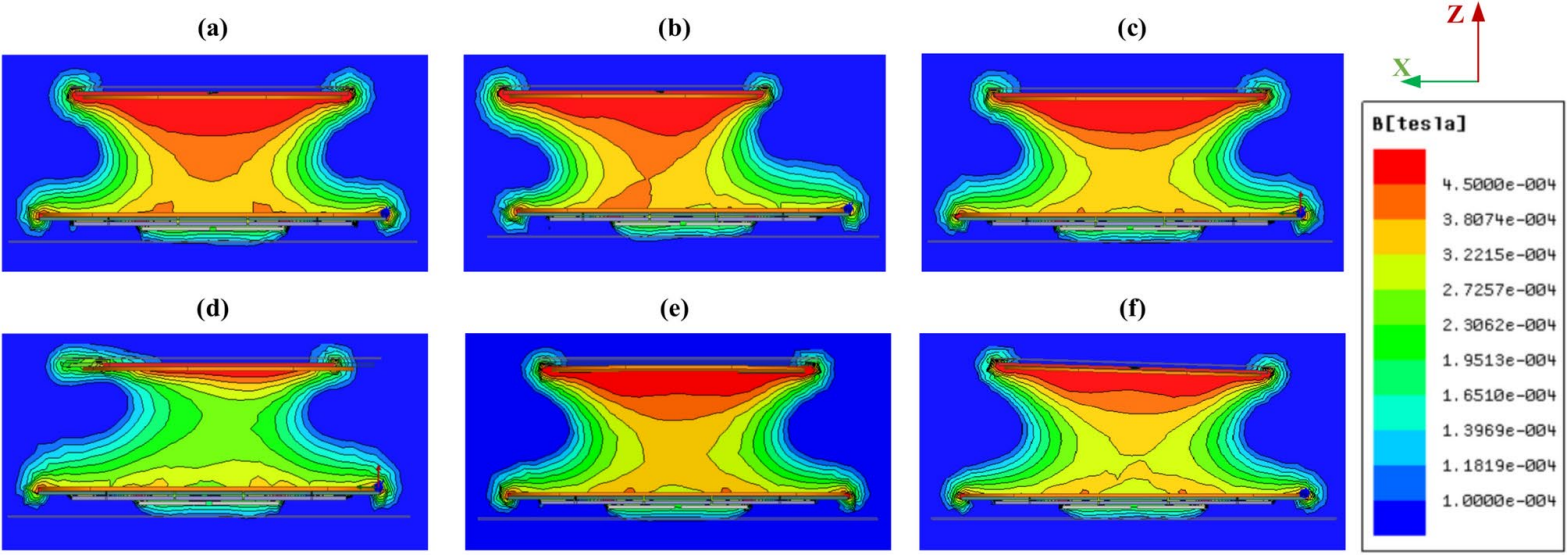
Distribution *of B* lines for CPT-CPR configuration, (**a**) ideal alignment, (**b**) ΔX = 75 mm, (**c**) ΔY = 100 mm, (**d**) *Yaw* = 10°, (**e**) *Roll* = 2°, and (**f**) *Pitch* = 2°.

**Fig. 15:**
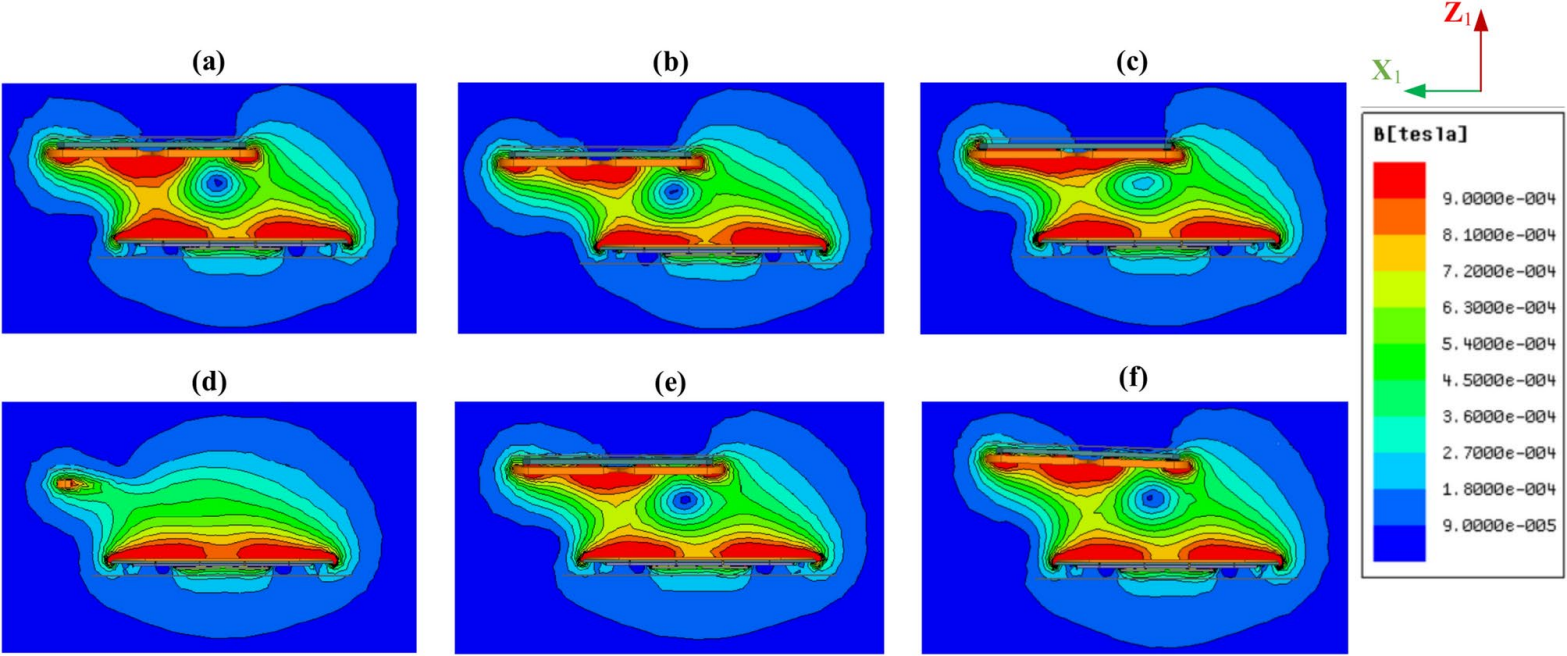
Distribution *of B* lines for CPT-DDPR configuration, (**a**) ideal alignment, (**b**) ΔX = 75 mm, (**c**) ΔY = 100 mm, (**d**) *Yaw* = 10°, (**e**) *Roll* = 2°, and (**f**) *Pitch* = 2°.

According to the SAE J2954 standard, the maximum safe magnetic field density (*B*) for charging applications involving living organisms and portable medical devices is set at 15 µT. To evaluate EMFs values under both perfect alignment and various misalignment conditions, the 3D-FEMs described in Sect.  2 were utilized. The study focused on the worst-case scenario (Z3-class), which presents the highest EMF levels due to the increased air gap distance. Figures 14 and 15 illustrate the distribution of magnetic flux density (*B*) for the CPT-CPR and CPT-DDPR configurations under both fully aligned and misaligned conditions, including linear and rotational misalignments. To avoid redundancy, only the CPT-CPR and CPT-DDPR configurations were displayed, as the DDPT-DDPR and DDPT-CPR configurations exhibit similar magnetic flux distributions. The results indicate that under different misalignment conditions, the distribution of the magnetic field varies. In the CPT-CPR and DDPT-DDPR configurations, the BB field lines remain concentrated in the space between the ground and vehicle pads. Conversely, in the DDPT-CPR and CPT-DDPR configurations, the BB field lines form a forked pattern, extending around the system and above the vehicle-side pad.

**Fig. 16:**
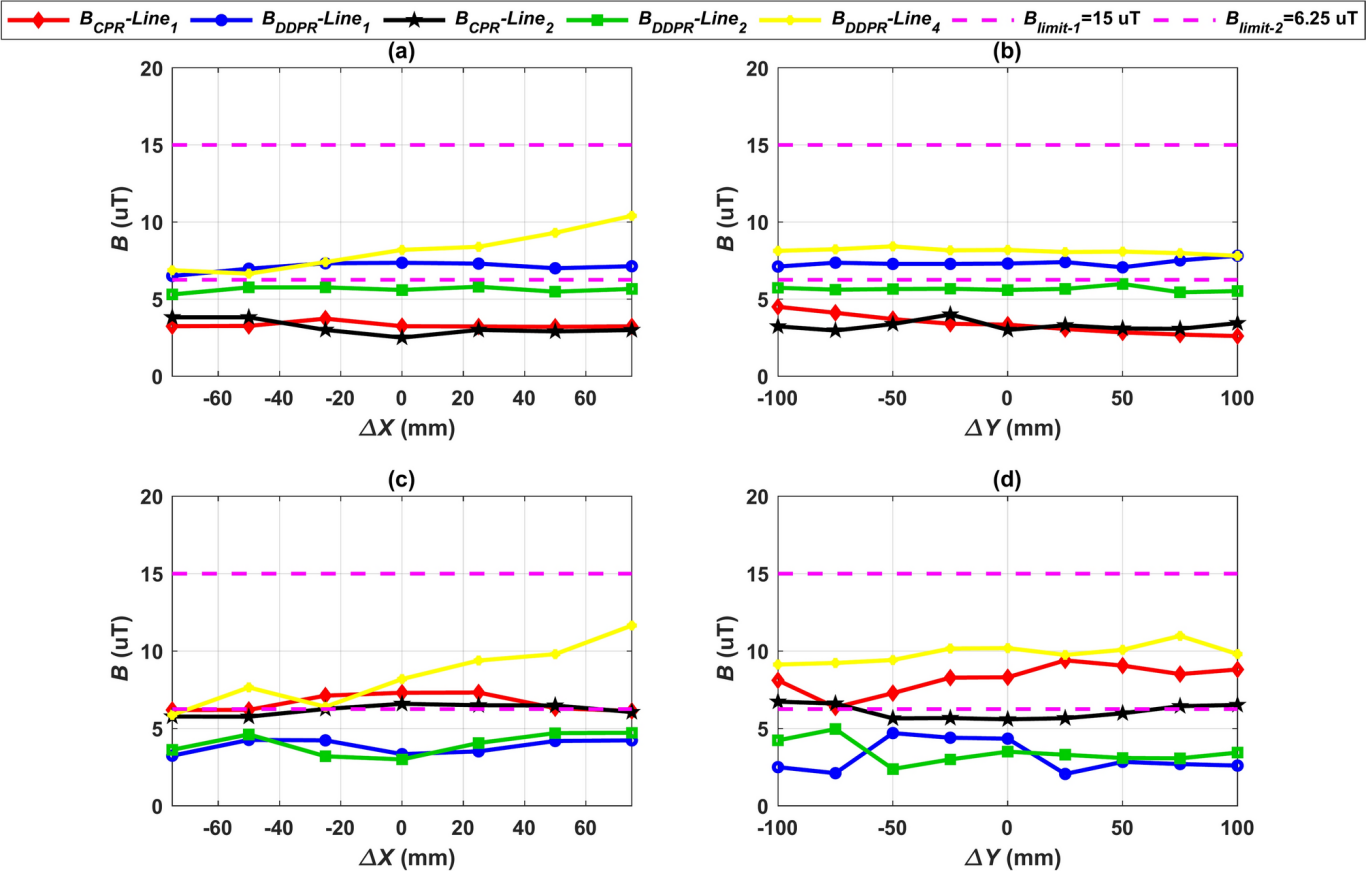
Distribution of *B* lines at the front, left, and right sides of the EV, denoted as line 1, line 2, and line 4 respectively, (**a**) *B* vs. *∆X* for CPT-CPR and CPT-DDPR configurations, (**b**) *B* vs. *∆Y* CPT-CPR and CPT-DDPR configurations, (**c**) *B* vs. *∆X* for DDPT-DDPR and DDPT-CPR configurations, and (**d**) *B* vs. *∆X* for DDPT-DDPR and DDPT-CPR configurations.

Four vertical lines, numbered 1 through 4, were used to measure the value of the magnetic field *B* (Fig. 12). The DDPT-DDPR and CPT-CPR configurations exhibit symmetry, which results in nearly identical EMF values for each of the two opposing lines (1 and 3) and (2 and 4). For this reason, only lines 1 and 2’s EMF results were shown. Since lines 1 and 3 of the DDPT-CPR and CPT-DDPR configurations exhibit the same outcomes due to symmetry in the Y1-axis, line 3’s outcomes can be ignored and only line 1’s results should be considered. However, as discussed in Sect. 2.4, there is a natural displacement for both the RT-DDR (± 180 mm) and DDT-RR (± 175 mm) models in the direction of the X1-axis. As a result, line 4 produces stronger EMFs than line 2. As illustrated in Fig. 16a,b, the magnetic field *B* values of the CPT-CPR and CPT-DDPR configurations are introduced in full alignment and linear misalignment conditions in the driving direction (X-axis) and Y-axis direction, respectively. While the magnetic field *B* values of the other configurations, DDPT-DDPR and DDPT-CPR, are expressed in Fig. 16c,d, respectively, in cases of linear misalignment in the driving direction and the Y-axis direction.

**Fig. 17:**
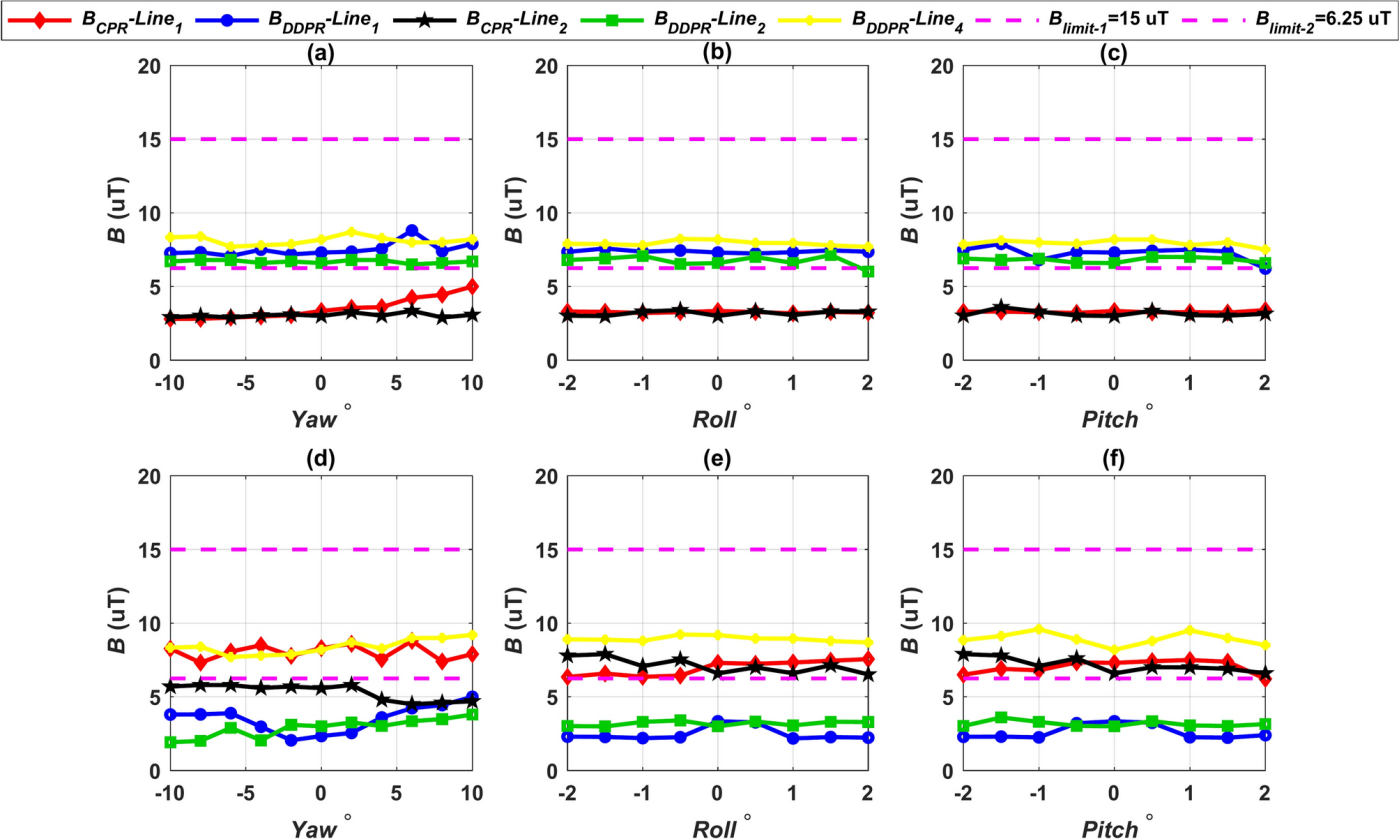
Distribution of *B* lines at the front, left, and right sides of the EV, denoted as line 1, line 2, and line 4 respectively, (**a**) *B* vs. *Yawº* for CPT-CPR and CPT-DDPR configurations, (**b**) *B* vs. *Rollº* CPT-CPR and CPT-DDPR configurations, (**c**) *B* vs. *Pitchº* CPT-CPR and CPT-DDPR configurations, (**d**) *B* vs. *Yawº* for DDPT-DDPR and DDPT-CPR configurations, (**e**) *B* vs. *Rollº* DDPT-DDPR and DDPT-CPR configurations, and (**f**) *B* vs. *Pitchº* DDPT-DDPR and DDPT-CPR configurations.

The configurations CPT-CPR and CPT-DDPR had their magnetic field *B* values calculated at various vertical lines in case of rotational misalignment conditions which are *Pitch* ^o^, *Roll*^o^, and *Yaw* ^o^, as illustrated in Fig. 17a–c. The *B* values for the DDPT-CPR and DDPT-DDPR configurations are presented in Fig. 17d–f. Under SAE j2954’s recommended maximum allowable limits of 15 µT, all configurations (CPT-CPR, CPT-DDPR, DDPT-DDPR, and DDPT-CPR) achieve a magnetic field density *B* below that limit. At varying misalignment conditions, the maximum value of these configurations is less than the safe maximum limit by 28.8%. Consequently, the measurement uncertainty is addressed, with a typical value of approximately 5%^73^. The CPT-CPR and DDPT-DDPR systems’ magnetic field *B* values at vertical lines 1 and 2 are extremely close to one another. The distribution of flux lines as illustrated in Figs. 14 and 15 is consistent with the higher values of the magnetic field *B* that the CPT-DDPR and DDPT-CPR configurations present at all lines in various misalignment states. When there is a natural offset present, line 4 provides the maximum magnetic field values in both full alignment and misalignment scenarios. Additionally, when the maximum misalignment happened at observation lines 5 and 6, the magnetic field *B* values were measured for all configurations (see Fig. 12). Table 11 lists the worst values (maximum misalignment) of the *B*. According to the information provided, it can be inferred that the magnetic field *B* values for every model being tested are significantly lower than the j2954’s recommended safe limit of 15 µT. As a result, it can be concluded that these configurations comply with the 2010 ICNIRP recommendations.

**Table 11:** Worst values of *B* in µTesla at line No. 5 and line No. 6 for all configurations.

Worst misalignment	Value	DDPT/DDPR		DDPT/CPR		CPT/CPR		CPT/DDPR	
		Line No. 5	Line No. 6	Line No. 5	Line No. 6	Line No. 5	Line No. 6	Line No. 5	Line No. 6
*∆X*	75 mm	0.75	3.78	1.32	7.21	0.72	3.23	1.29	6.66
*∆Y*	100 mm	0.65	3.67	1.24	7.33	0.69	3.44	1.09	6.52
Yaw	10°	1.05	3.56	1.68	6.98	0.99	3.08	1.70	6.70
Roll	2°	0.84	3.25	1.54	6.24	0.78	3.29	1.43	6.02
Pitch	2°	0.68	3.08	1.87	6.77	0.73	3.15	1.32	6.60

The 1998 ICNIRP guidelines were followed during the testing of the charging systems suggested in this study in order to increase safety. These recommendations are more cautious than the 2010 ICNIRP because they set the maximum safe limit for stray magnetic fields at 6.25 µT. The 1998 ICNIRP guidelines, which serve as the safe limit for stray magnetic fields, have been legally enforced in the majority of European countries^74,75^. At different misalignments, all configurations were assessed at the Z3-class in relation to the 6.25 µT limit. Figures 16 and 17 as well as Table 11 demonstrate a good agreement between the CPT-CPR and DDPT-DDPR models and the safe limit of 6.25 µT. Considering the measurement uncertainties, these two models produced a magnetic field that was 25% lower than the maximum value. The magnetic field shown by the DDPT-CPR and CPT-DDPR models is 46.35% higher than the maximum value.

Consequently, since the maximum value of *B* is below the boundaries permitted by the ICNIRP guidelines in both its 1998 and 2010 versions, it can be mentioned that the two configurations, CPT-CPR and DDPT-DDPR, are completely compliant with the limits. Furthermore, *B* values obtained by the DDPT-CPR and CPT-DDPR configurations are below the allowable limits as stated in the 2010 ICNIRP. Instead, they do not adhere to the 1998 ICNIRP limits, where the *B* values exceed the 6.25 µT maximum allowable safe limit. Thus, it can be concluded that the 1998 ICNIRP guidelines are incompatible with the DDPT-CPR and CPT-DDPR configurations. This indicates that further research is necessary to determine whether the guidelines are compatible within its bounds.

By reducing the system’s power consumption, magnetic field levels can be decreased. In order to determine which power levels are compatible with the maximum value of 6.25 µT, an analysis of the CPT-DDPR and DDPT-CPR configurations was done. Up to a 5% reduction in magnetic field density *B* (less than 6.25 µT), the level of output power is adjusted. Table 12 contains the maximum *B* values for both configurations, along with the corresponding power level and efficiency at various misalignments. Remarkably, power must be reduced by a small percentage (less than 10%) in order to bring the systems into compliance with the ICNIRP guidelines from 1998. Because conduction losses and leakage electromagnetic fields are reduced in the de-rated configurations, transmission efficiency is slightly increased.

**Table 12:** De-rated configurations CPT-DDPR and DDPT-CPR.

Configuration	*B*_*-worst*_		*P*_*o*_			η	
	Original value	De-rated	Original value	De-rated	% Change	Original value	De-rated
CPT-DDPR	10.40 µT	5.85 µT	10.25 kW	9.61 kW	6.24**%**	92.34**%**	92.72**%**
DDPT-CPR	11.65 µT	4.73 µT	10.46 kW	9.54 kW	8.79**%**	94.23**%**	94.58**%**

### Leakage electric field assessment

**Fig. 18:**
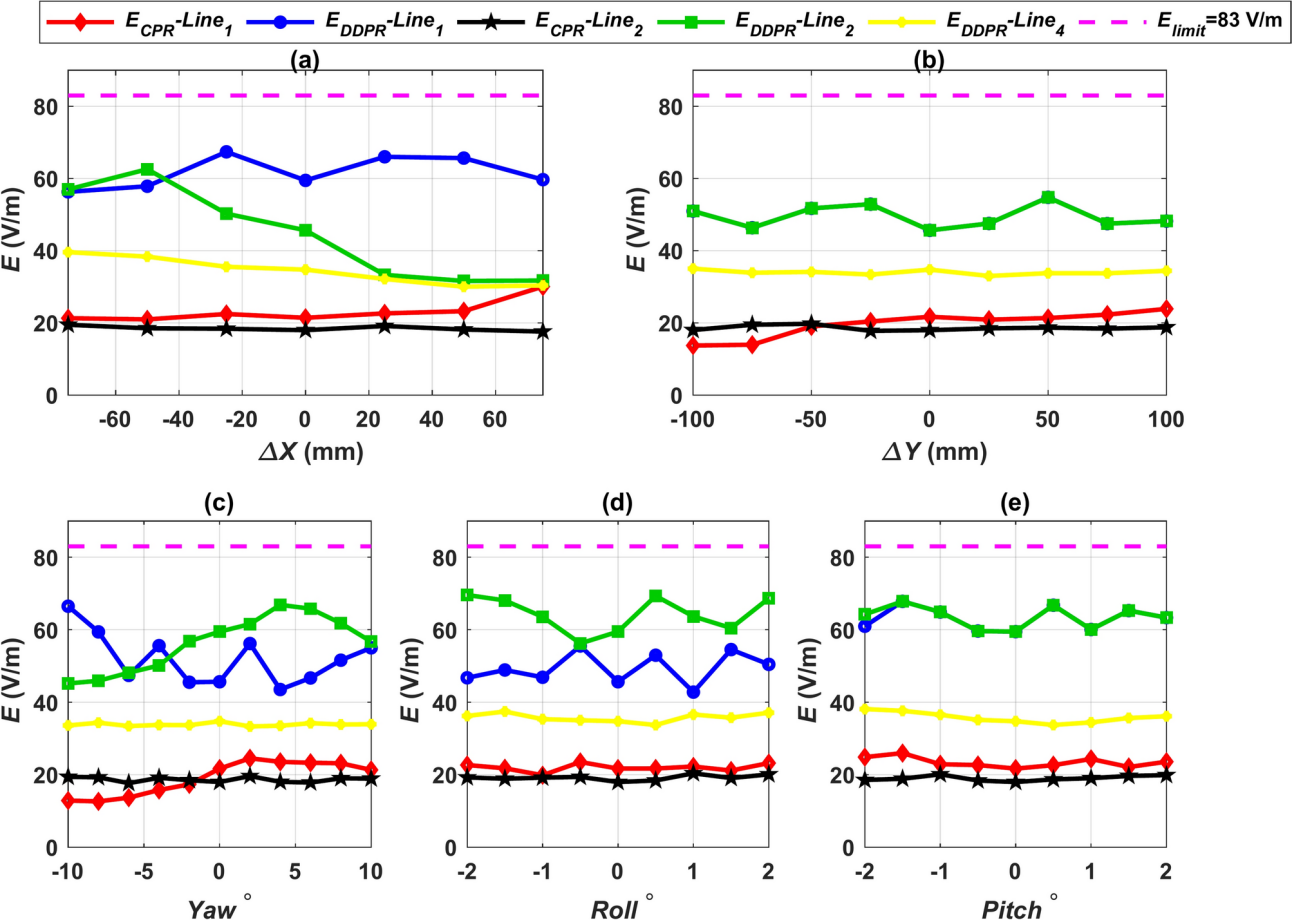
Distribution of *E* lines for CPT-CPR and CPT-DDPR configurations at the front, left, and right sides of the EV, denoted as line 1, line 2, and line 4 respectively, (**a**) *E* vs. *∆X*, (**b**) *E* vs. *∆Y*, (**c**) *E* vs. *Yawº*, (**d**) *E* vs. *Rollº*, and (**e**) *E* vs. *Pitchº*.

**Fig. 19:**
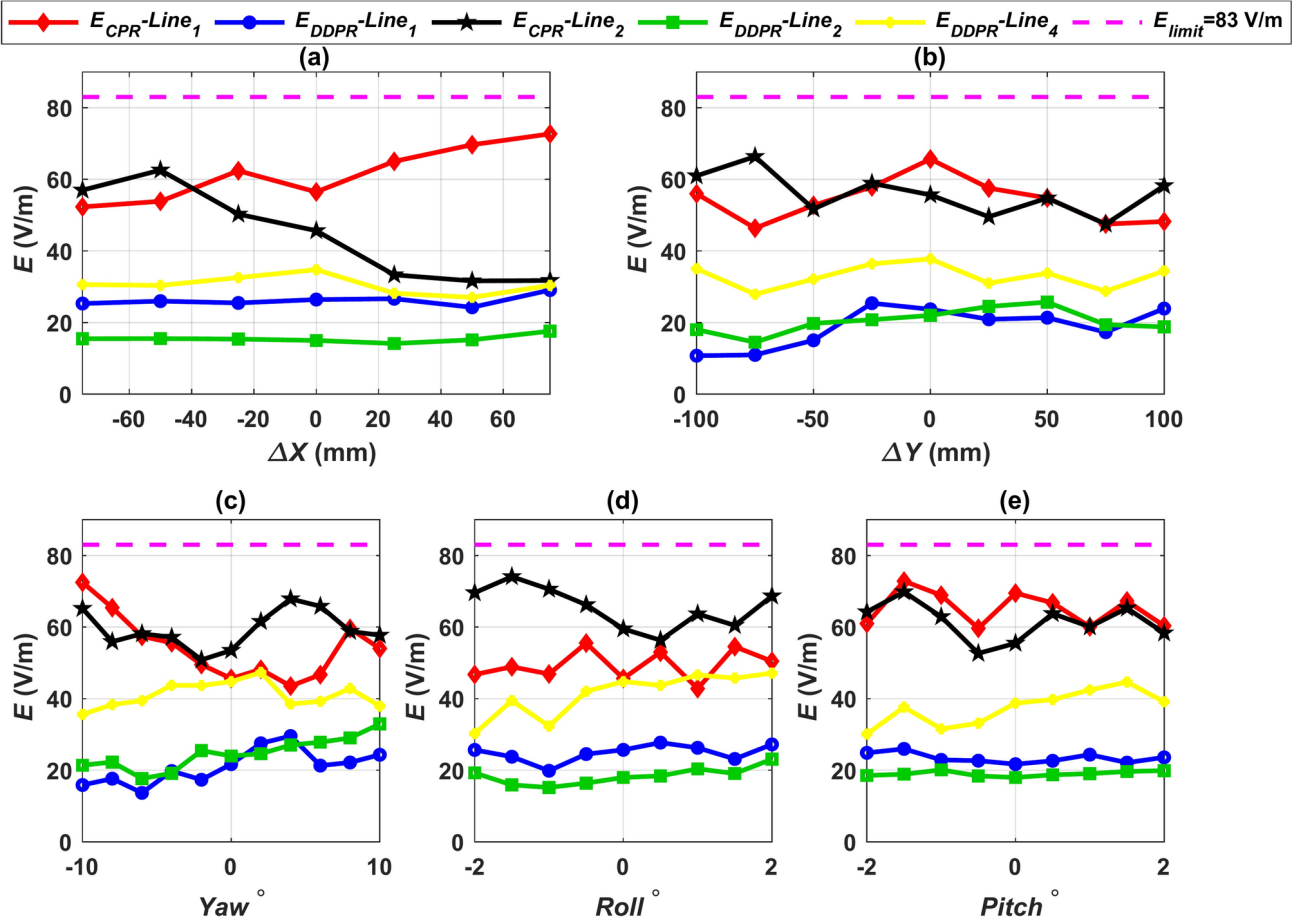
Distribution of *E* lines for DDPT-DDPR and DDPT-CPR configurations at the front, left, and right sides of the EV, denoted as line 1, line 2, and line 4 respectively, (**a**) *E* vs. *∆X*, (**b**) *E* vs. *∆Y*, (**c**) *E* vs. *Yawº*, (**d**) *E* vs. *Rollº*, and (**e**) *E* vs. *Pitchº*.

The electric field surrounding the charging system must not exceed the safe limits specified in the two versions of the ICNIRP guidelines in addition to the safe limits pertaining to the magnetic field. The safe limit for electric field intensity (*E*) was 87 V/m in the 1998 ICNIRP guidelines, but the 2010 guidelines set a maximum safe value of 83 V/m^75^. At various linear and angular misalignments, the electric field intensity (*E*) for each design was measured at the same test lines as the magnetic field. By extracting the transmitting and receiving coil voltages from the MATLAB Simulink circuit, one can estimate the electric field intensity *E*. These values are then input into the corresponding FEMs. Lines 1, 2, and 4 in Figs. 18 and 19 and lines 5 and 6 in Table 13 display the *E* values for all configurations at the worst airgap distance (Z3-class) and under different misalignment conditions. The substantial disparity in the voltages of the ground and car side coils is the reason why the electric field *E* values for the two configurations DDPT-DDPR and DDPT-CPR are, in every state of misalignment, higher than those for the two configurations CPT-CPR and CPT-DDPR. All *E* values are still, nevertheless, below the 83 V/m suggested safe limits. We can conclude that every system put forth in this research is completely compliant with the safe limits for electric field *E* that are outlined in the ICNIRP guidelines from 1998 and 2010.

**Table 13:** Worst values of E in V/m at line No. 5 and line No. 6 for all configurations.

Worst misalignment	Value	DDPT/DDPR		DDPT/CPR		CPT/CPR		CPT/DDPR	
		Line No. 5	Line No. 6	Line No. 5	Line No. 6	Line No. 5	Line No. 6	Line No. 5	Line No. 6
*∆X*	75 mm	2.53	7.42	52.02	66.45	2.20	6.86	47.45	62.13
*∆Y*	100 mm	1.99	7.98	54.65	70.24	1.81	7.70	43.47	65.35
Yaw	10^o^	2.43	8.54	53.25	74.32	2.07	7.36	48.35	73.25
Roll	2°	2.66	8.35	58.01	69.80	2.31	7.99	48.99	68.74
Pitch	2°	2.98	8.47	57.62	69.45	2.78	7.59	47.75	67.87

## Conclusion and future work

This study conducts a thorough safety evaluation of various configurations used for charging EVs, focusing on the two most widely adopted pad configurations in an IPT system. Both CP and DDP pads were tested when placed on the ground side. By utilizing DDPT and CPT as universal transmitters, the study assessed leakage EMFs safety across different receiver configurations (CPR and DDPR). The development of 3D-FEMs for the four configurations (DDPT-DDPR, DDPT-CPR, CPT-CPR, and CPT-DDPR) was carried out following the SAE J2954 guidelines. FEMs and MATLAB Simulink circuits were implemented for safety analysis and validation. Safety assessments of these four charging configurations were conducted in accordance with the ICNIRP guidelines from 1998 and 2010. The results revealed that while all configurations complied with the magnetic field leakage limits specified in the 2010 ICNIRP guidelines, the magnetic flux density (*B*) in the DDPT-CPR and CPT-DDPR configurations exceeded the permissible limits set by the 1998 ICNIRP guidelines. However, reducing the power levels of these configurations ensured their compliance with the 1998 ICNIRP standards. Additionally, all four configurations met both the 1998 and 2010 ICNIRP guidelines regarding electric field exposure, with electric field (*E*) values remaining below the safe threshold of 83 V/m. In summary, the findings confirm that all four configurations can operate efficiently while maintaining the required safety levels for both pacemakers and living organisms, effectively transferring the intended output power.

While the study confirms that both double-D pad (DDPT) and circular pad (CPT) configurations comply with ICNIRP safety limits, additional safety considerations can be explored, particularly regarding long-term exposure and other radiation effects.

- For long-term exposure considerations:
  - Current ICNIRP guidelines address short-term exposure, but long-term exposure to low-level EMFs is under-researched.
  - Potential biological effects, such as cellular stress and neurological impacts, require further investigation.
  - Cumulative exposure in public spaces or homes may pose unknown health risks.
- For other types of radiation and safety concerns:
  - Electromagnetic Interference (EMI): Potential interference with medical devices and vehicle electronics; further exploration of EMI shielding and compliance with ISO 7637 and CISPR standards is needed.
  - Higher-Frequency EMFs: as WPT systems evolve to higher frequencies, new safety assessments will be necessary due to different exposure mechanisms.
- Carry out a thorough experimental analysis and testing of the suggested design.
- Investigate the compatibility and interoperability between a solenoid receiver (SR) and the global CPT and DDPT utilized in this manuscript.
- Investigate the impact of flush and underground transmitter pad installations on the interoperability, safety, and effectiveness of static inductive systems.
- Investigate the impact of material properties, coil configurations, air gap variations, and environmental conditions on all proposed design’s safety.
- Investigate Electromagnetic Compatibility (EMC) issues for all systems.

## Human and animal rights

This article does not contain any studies with animals performed by any of the authors.

## Data Availability

The data used to support the findings of this study are available from the corresponding author upon request.
